# Multiharmonic Algorithms for Contrast-Enhanced Ultrasound

**DOI:** 10.1007/s10915-026-03247-2

**Published:** 2026-03-24

**Authors:** Vanja Nikolić, Teresa Rauscher

**Affiliations:** 1 Department of Mathematics, Radboud University, Heyendaalseweg 135, 6525 AJ Nijmegen, The Netherlands; 2 Department of Mathematics and Scientific Computing, University of Graz, Heinrichstraße 36, A-8010 Graz, Austria

**Keywords:** Nonlinear acoustics, Contrast-enhanced ultrasound, Microbubbles, Multiharmonic expansions, Westervelt’s equation, Helmholtz equation, Iterative algorithms, Primary 35L05, 35L72, 34A34, 35J05

## Abstract

Harmonic generation plays a crucial role in contrast-enhanced ultrasound, both for imaging and therapeutic applications. However, accurately capturing these nonlinear effects is computationally demanding when using traditional time-domain approaches. To address this issue, we develop algorithms based on a time discretization that uses a multiharmonic Ansatz applied to a model that couples the Westervelt equation for acoustic pressure with a volume-based approximation of the Rayleigh–Plesset equation for the dynamics of microbubble contrast agents. We first rigorously establish the existence of time-periodic solutions for this Westervelt-ODE system. We then derive a multiharmonic representation of the system under time-periodic excitation and develop iterative algorithms that rely on the successive computation of higher harmonics assuming either real-valued or complex-valued solution fields. In the real-valued setting, we characterize the approximation error in terms of the number of harmonics and a contribution arising from the fixed-point iteration. Finally, we investigate these algorithms numerically and illustrate how the number of harmonics and the presence of microbubbles influence the propagation of acoustic waves.

## Introduction

Contrast-enhanced ultrasound has become an important tool in biomedical applications, with gas-filled microbubbles being used to improve both diagnostic and therapeutic procedures. While ultrasound propagation can be described by linear acoustic models in the small-amplitude regime, nonlinear effects arise at higher acoustic pressures, leading to the generation of higher harmonics in the frequency domain. These nonlinearities are further amplified by microbubble contrast agents, which exhibit strongly nonlinear oscillatory behavior when exposed to ultrasound waves. This delicate back-and-forth interaction is beneficial for improving resolution in imaging but also for enhancing therapeutic treatments, such as targeted drug delivery; see, for example, [12, 18, 37] for details. With the rise in the number of applications of contrast-enhanced ultrasound, accurate modeling and efficient simulation in this context have also become prominent research topics; see, e.g., [6, 10, 28, 39], and the references given therein.

The present work builds upon [33], where time-domain mathematical models for ultrasound contrast imaging with microbubbles based on a nonlinear acoustic wave equation coupled to a Rayleigh–Plesset-type ODE in a continuum (effective-medium) description of the bubbly mixture have been derived and investigated in terms of local well-posedness and numerical simulations. As noted in [33], a major computational challenge when simulating such systems stems from different time scales on which the wave equation and ODE (nonlinearly) evolve. As a result, straightforward numerical approaches for solving such systems demand using prohibitively small time steps. In this work, we approach this issue by developing *multiharmonic* algorithms for time-periodic solution fields, which offer a potentially more efficient modeling alternative by making use of harmonic expansions; see, e.g., [3, 4], where multiharmonic ideas have been explored for problems arising in electromagnetism and, e.g., [15, 21, 22, 34], where they have been developed for single-physics acoustic models. Instead of resolving every oscillation in the space-time domain, these methods decompose the field into a sum of harmonics, which can be obtained as solutions of suitable (in our case) Helmholtz problems and algebraic equations. These methods are especially promising for simulating real-time ultrasound applications in which the number of harmonics used can be relatively low.

As the starting time-domain model of contrast-enhanced ultrasound we employ the following coupled nonlinear wave-ODE system:

1 $$\begin{aligned} \boxed { \begin{aligned}&\ \ p_{tt} - c^2 \Delta p - b \Delta p_t = \eta (p^2)_{tt} + c^2 \rho _0 n_0(x) v_{tt}+ h(x,t) \quad  &   \text {in } \Omega \times (0,T), \\&\ \ v_{tt} + \delta \omega _0 v_t + \omega _0^2 v = \zeta v^2 + \xi (2v v_{tt}+ v_t^2)- \mu p \quad  &   \text {in } \Omega \times (0,T), \\ \end{aligned} } \end{aligned}$$

consisting of the damped Westervelt equation [40] for the acoustic pressure $p=p(x,t)$ (that is, fluctuations in the background pressure) and an ODE pointwise a.e. in space for the volume variation $v=v(x,t)$ of microbubbles. The system in (1) models the interaction of acoustic pressure waves with oscillations of microbubbles.

### Modeling Background

In the Westervelt equation, $c>0$ denotes the (constant) speed of sound in the medium, $b>0$ the diffusivity of sound, so that the term $-b \Delta p_t$ introduces strong damping, and $\rho _0>0$ the mass density of the mixture at equilibrium. The nonlinearity coefficient is given by $\eta = \frac{\beta _a}{\rho _0 c^2}$, where $\beta _a$ is the nonlinearity parameter in the medium. The source of the pressure waves is provided in part through a contribution due to bubble oscillations, modeled by the term $c^2 \rho _0 n_0 v_{tt}$, where $n_0=n_0(x)$ is the bubble number density at equilibrium, and by the source function $h=h(x,t)$. We allow $n_0$ to vary in space (that is, we assume that $n_0 \in L^\infty (\Omega )$), as this setting is relevant for imaging applications; see, e.g., [25]. Moreover, $n_0$ and *h* regulate the strength of the acoustic source and will be assumed to be small enough in the well-posedness analysis; see Theorem 1 and the discussion in Section 2.3 on the physical meaning of the smallness assumption.

The ODE in (1) captures nonlinear harmonic oscillations driven by the acoustic pressure via the term $- \mu p$. The total microbubble volume is given by $V= v_0 + v$, where $v_0$ is the equilibrium volume. The coefficient $\delta = \frac{4 \nu }{\omega _0 R_0^2}$ is the viscous damping coefficient and $\omega _0 = \sqrt{\frac{3 \kappa P_0}{\rho _0 R_0^2}}$ the natural frequency, where $R_0$ is the bubble radius at equilibrium volume $v_0$ (that is, $v_0 = \frac{4 \pi }{3} R_0^3$), $\kappa$ the adiabatic exponent and $P_0$ the ambient pressure in the mixture (so that the total pressure is $P_0 +p$). The coefficients appearing on the right-hand side of the ODE are given in terms of equilibrium values by $\mu = \frac{4 \pi R_0}{\rho _0}$, $\zeta = \frac{(\kappa + 1) \omega _0^2}{2 v_0}$, and $\xi = \frac{1}{6 v_0}$. As discussed in [17, Ch. 5], the ODE in (1) can be seen as a volume-based approximation of the following Rayleigh–Plesset equation:

$$\begin{aligned} \rho _0 \left[ R R_{tt} + \tfrac{3}{2} R_t^2 \right] = p_b - 4 \nu \frac{R_t}{R} - p, \end{aligned}$$

where $\nu$ is the kinematic viscosity and $p_b$ a constant pressure contribution, which can be derived using the relation $V= \frac{4 \pi }{3} R^3$ and the adiabatic gas law $\frac{p_b}{P_0} = \left( \frac{v_0}{V}\right) ^{\kappa }$. Introducing the volume variable and expanding the resulting expression about the equilibrium radius $R_0$, while neglecting higher-order terms, yields a simplified ODE for the volume perturbation *v*. A detailed derivation of this approximation is provided in [35, Sec. 2.3]. Reformulating the dynamics in terms of *v* instead of *R* removes singular terms and results in a more manageable nonlinear structure. Since pressure perturbations couple more directly to volume oscillations, the volume-based formulation is also better suited for harmonic expansions. In contrast, a harmonic expansion in terms of *R* is expected to lead to higher-order interactions that complicate the isolation of harmonic components due to the cubic dependence of volume on radius.

We equip (1) with absorbing-type boundary conditions of the following form:

$$\begin{aligned} \beta p_t + \gamma p + \nabla p \cdot \boldsymbol{n}= 0 \quad \text { on } \partial \Omega \times (0, T), \quad \beta ,\, \gamma >0, \end{aligned}$$

and we are interested in time-periodic solutions that satisfy

$$\begin{aligned} \begin{aligned} p(0)=p(T), \qquad p_t(0)=p_t(T) \qquad \text { in } \Omega , \\ v(0)=v(T), \qquad v_t(0)=v_t(T) \qquad \text { in } \Omega , \end{aligned} \end{aligned}$$

which corresponds to steady-state oscillatory (stable cavitation) regimes rather than transient cavitation. Although in the existence analysis we do not require the source *h* to be time periodic, for developing multiharmonic algorithms, we assume that *h* represents the second time derivative of a *T*-periodic function *g*; see Section 3 for details.

### Main Contributions

The overall purpose of the present work is to establish multiharmonic approximation approaches for time-domain systems in the form of (1), that is, systems that incorporate both nonlinear acoustic propagation and nonlinear microbubble oscillations. To approximate the problem we employ a cut-off Fourier series for the pressure and volume fields:

2 $$\begin{aligned} \begin{aligned} p \approx p^N= \mathfrak {R}\left\{ \sum _{m=0}^{N} \exp (\imath m \omega t) p_m^N(x)\right\} , \quad v \approx v^N= \mathfrak {R}\left\{ \sum _{m=0}^{N} \exp (\imath m \omega t) v_m^N(x)\right\} , \end{aligned} \end{aligned}$$

with $\omega = \dfrac{2 \pi }{T}$. In addition to rigorously considering the setting of real-valued pressure-volume fields, we also discuss multiharmonic algorithms derived from complex-valued fields (that is, from dropping the $\mathfrak {R}$ operator in (2)). This approach is sometimes adopted in electromagnetism (see, e.g., [9, 41]) and it has been discussed in [21] for the de-coupled Westervelt equation. Here, it results in simplified algorithms, which are, however, only formally investigated; see Section 5 for details. Toward reaching the overall goal of the work, our main contributions pertain to

- rigorously establishing the existence of time-periodic solutions for the Westervelt-ODE system in (1);
- deriving cutoff multiharmonic approximations of the system under time-periodic excitation for computing $(p^N_m, v^N_m)$;
- developing and analyzing *linearized* multiharmonic cut-off algorithms for computing $$(p^N_m, v^N_m) $$.

**Fig. 1:**
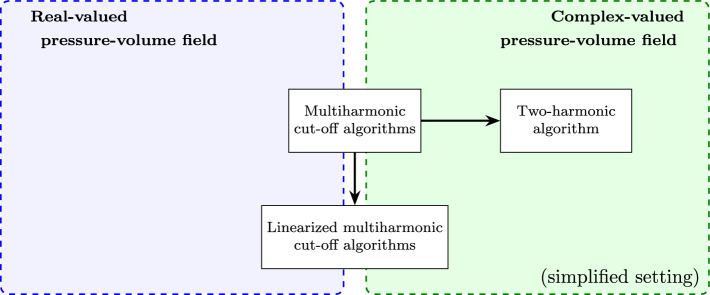
Overview of multiharmonic algorithms in this work

In particular, we characterize the error of linearized multiharmonic algorithms for real-valued fields in terms of the number of harmonics and a contribution due to the fixed-point iteration; see Theorem 2. For convenience, an overview of algorithms investigated in this work is provided in Figure 1.

### Novelty and Related Work

To the best of our knowledge, this is the first work to rigorously develop multiharmonic approaches for coupled nonlinear wave-ODE systems of this type, providing both well-posedness theory and numerical analysis. An approach to second harmonic generation for a linearization of (1) with $\eta =0$ and $b=0$ in the wave equation has been formally set up in [17, Ch. 5] without convergence guarantees.

Rigorous multiharmonic studies have been performed on single-physics equations. Existence of periodic solutions of nonlinear acoustic models, including the Westervelt equation, has been investigated rigorously in [7, 8, 21–23, 34]. An iterative multiharmonic algorithm for the Westervelt equation has been proposed and rigorously studied in [34]. A numerical algorithm based on using the complex Fourier Ansatz for the de-coupled Westervelt equation and a boundary element approach for the resulting Helmholtz problems has been developed and investigated in [15]. In the context of electromagnetism, a multiharmonic treatment of the quasi-stationary Maxwell problem has been developed and rigorously analyzed in [3]; numerical simulation aspects are discussed in [4]. These results, however, do not address the coupling to microbubble/ODE dynamics.

Periodic solutions of Rayleigh–Plesset-type equations that do not incorporate modeling of the acoustic propagation have been rigorously investigated in the literature; we refer to, for example, [16, 42] and [38, Ch. 9], and the references provided therein. We mention in passing that the mathematical literature on non-periodic models in nonlinear acoustics of non-bubbly media is quite rich; see, e.g., [1, 11, 20, 24] and the references provided therein.

### Organization of the Paper

The remainder of the exposition is organized as follows. In Section 2, we determine sufficient conditions for the well-posedness of the time-periodic boundary value problem for the Westervelt-ODE system (1). In Section 3, we derive a multiharmonic cut-off representation of the system and propose a linearization which results in a simplified setting for computing Fourier coefficients in (2). In Section 4, we characterize the error and prove the convergence of the proposed linearized multiharmonic scheme to the solution of the Westervelt-ODE system as the number of harmonics *N* tends to $\infty$. The main theoretical result of the section is contained in Theorem 2. In Section 5, we discuss multiharmonic algorithms resulting from complex solutions fields. Finally, in Section 6, we investigate and compare the introduced algorithms.

## Existence of Time-Periodic Solutions

In this section, we establish the basis for the numerical analysis by investigating the existence and uniqueness of periodic solutions for the coupled PDE-ODE system. More precisely, given $T>0$ and a bounded domain $\Omega \subset \mathbb {R}^d$, where $d \in \{2,3\}$, we study the problem:

3 $$\begin{aligned} \left\{ \begin{aligned} \  &p_{tt}- c^2 \Delta p - b \Delta p_t= \eta (p^2)_{tt}+c^2 \rho _0 n_0(x) v_{tt}+ h \quad  &   \text {in } \Omega \times (0,T),\\&\beta p_t+ \gamma p + \nabla p \cdot \boldsymbol{n}= 0  &   \text {on } \partial \Omega \times (0,T), \\&p(0) = p(T), \ p_t(0) = p_t(T)  &   \text {in } \Omega , \\&v_{tt}+ \delta \omega _0 v_t+ \omega _0^2 v = \zeta v^2 + \xi (2v v_{tt}+ v_t^2)- \mu p \quad  &   \text {in } \Omega \times (0,T),\\&v(0) = v(T), \ v_t(0) = v_t(T)  &   \text {in } \Omega . \end{aligned} \right. \end{aligned}$$

The analysis will be based on successive approximations of the system, for which knowledge of linear time-periodic ODE and wave problems will be very helpful. We thus discuss those results next.

**Notation**. Below we use $lhs \lesssim rhs$ to denote $lhs \le C \cdot rhs$, where $C>0$ is a generic constant. When writing norms in Bochner spaces, we omit the temporal domain (0, *T*). For example, $\Vert \cdot \Vert _{L^p(L^q(\Omega ))}$ denotes the norm in $L^p(0,T; L^q(\Omega ))$.

### Auxiliary Existence Results for Linear Time-Periodic Problems

To set up the local well-posedness analysis of (3), we first state two separate results on the well-posedness of an ODE (describing damped oscillations with forcing) and a linear time-periodic wave problem.

#### Lemma 1

Let $T>0$ and $f \in L^2(0,T; L^\infty (\Omega ))$. Furthermore, let $\delta$, $\omega _0>0$. Then, the periodic ODE problem

$$\begin{aligned} \left\{ \begin{aligned}&v_{tt}+ \delta \omega _0 v_t+ \omega _0^2 v = f  &   \text {a.e.\ in } \Omega \times (0,T),\\&v(0) = v(T), \ v_t(0) = v_t(T)  &   \text {a.e.\ in } \Omega , \end{aligned} \right. \end{aligned}$$

has a unique solution

$$\begin{aligned} \begin{aligned} v \in \mathcal {X}_v=\{v \in H^2(0,T; L^\infty (\Omega )): \, v(0)=v(T), \, v_t(0)= v_t(T) \ \text {a.e.}\} \end{aligned} \end{aligned}$$

that satisfies

$$\begin{aligned} \begin{aligned} \Vert v\Vert _{\mathcal {X}_v} :=\Vert v\Vert _{L^\infty (L^\infty (\Omega ))}+ \Vert v_t\Vert _{L^\infty (L^\infty (\Omega ))}+\Vert v_{tt}\Vert _{L^2(L^\infty (\Omega ))} \lesssim \Vert f\Vert _{L^2(L^\infty (\Omega ))}. \end{aligned} \end{aligned}$$

#### Proof

The existence and uniqueness can be established using Floquet theory (see, for example [36] and [14, Ch. 3]) as the only solution of the homogeneous problem is zero due to the fact that $\delta \omega _0>0$. The details are provided in Appendix A for completeness. $\square$

#### Proposition 1

(see [34, Theorem 2.1]) Let $T>0$, $\Omega \subset \mathbb {R}^d$, where $d \in \{2,3\}$, be a bounded domain with $C^{1,1}$ boundary, and $\beta ,\,\gamma >0$, *c*, $b>0$. Let $h\in L^2(0,T;L^2(\Omega ))$. Then there exists a unique (weak) solutionof the time-periodic boundary-value problem

4 $$\begin{aligned} \left\{ \begin{aligned}&p_{tt}- c^2 \Delta p - b \Delta p_t= h\quad  &   \text {in } \Omega \times (0,T),\\&\beta p_t+ \gamma p + \nabla p \cdot \boldsymbol{n}= 0  &   \text {on } {\partial \Omega \times (0,T)}, \\&p(0) = p(T), \ p_t(0) = p_t(T)  &   \text {in } \Omega . \end{aligned} \right. \end{aligned}$$

The solution satisfies

$$\begin{aligned} \begin{aligned} \Vert p\Vert _{\mathcal {X}_p}:=&\, ||p||_{L^2(H^2(\Omega ))} + ||p_t||_{L^2(H^{3/2}(\Omega ))}+\Vert p_{tt}\Vert _{L^2(L^2(\Omega ))} + \Vert \Delta p_t\Vert _{L^2(L^2(\Omega ))} \\&\quad + \Vert \nabla p \cdot \boldsymbol{n}\Vert _{H^1(L^2(\partial \Omega ))} \\ \le&\,{ C(b, \gamma , c, \beta , T, \Omega ) ||h||_{ L^2(0,T;L^2(\Omega ))}}, \end{aligned} \end{aligned}$$

for some constant $C=C(b, \gamma ,c,\beta , T, \Omega ) > 0$, independent of *h*.

We note that having $\gamma >0$ in (4) is crucial for uniqueness. Furthermore, having strong damping (that is, $-b \Delta p_t$ with $b>0$) in the wave equation contributes to its parabolic-like character, and is heavily exploited in the proof of the above statement. We also note that the functions *f* and *h* do not have to be time periodic for the well-posedness results in this section, although we will make that assumption on the acoustic source in Section 3 to develop (iterative) multiharmonic algorithms.

### Analysis of the Westervelt-ODE System

Equipped with the previous results on linear problems, we are now ready to prove the well-posedness of the coupled pressure-volume problem. To state it, we introduce

$$\begin{aligned} \begin{aligned} \mathbb {B}_{r_p, r_v}= \left\{ (p, v) \in \mathcal {X}_p\times \mathcal {X}_v:\, \Vert p\Vert _{\mathcal {X}_p} \le r_p, \ \Vert v\Vert _{\mathcal {X}_v} \le r_v\right\} , \end{aligned} \end{aligned}$$

where $r_p>0$ and $r_v>0$ will be set as small enough in the course of the proof below. The proof will have constructive nature using successive approximations.

#### Theorem 1

Let $T>0$, $\Omega \subseteq \mathbb {R}^d$, $d \in \{2,3\}$, be open, bounded, connected, with $C^{1,1}$ boundary, $\beta$, $\gamma$, *c*, *b*, $\delta$, $\omega _0>0$ and $\eta$, $\mu$, $\zeta$, $\xi \in \mathbb {R}$, $n_0 \in L^\infty (\Omega )$, $h \in L^2(0,T; L^2(\Omega ))$. Then there exist $\delta _p>0$ and $\delta _v>0$, such that if

5 $$\begin{aligned} ||h||_{L^2(0,T;L^2(\Omega ))} \le \delta _p\quad \text {and} \quad \Vert {n_0}\Vert _{L^\infty (\Omega )} \le \delta _v, \end{aligned}$$

then there exist radii $r_p>0$ and $r_v>0$, such that problem (3) has a unique solution $(p, v) \in \mathbb {B}_{r_p, r_v}$.

#### Proof

As announced, the proof follows by constructing the solutions using successive approximations. Let $r_p$, $r_v>0$. We take $(p^{(0)}, v^{(0)}) \in \mathbb {B}_{r_p, r_v}$ and set up successive approximations using the system given by

6a $$\begin{aligned} \left\{ \begin{aligned} \  &p^{(N)}_{tt} - c^2 \Delta p^{(N)}- b \Delta p^{(N)}_t = \eta ((p^{(N-1)})^2)_{tt}+c^2 \rho _0 n_0 v^{(N)}_{tt} + h \,  &   \text {in } \Omega \times (0,T),\\&\beta p^{(N)}_t + \gamma p^{(N)}+ \nabla p^{(N)}\cdot \boldsymbol{n}= 0  &   \text {on } \partial \Omega \times (0,T), \hspace{-0.28cm}\\&p^{(N)}(0) = p^{(N)}(T), \ p^{(N)}_t(0) = p^{(N)}_t(T)  &   \text {in } \Omega , \end{aligned} \right. \end{aligned}$$

and

6b $$\begin{aligned} \left\{ \begin{aligned}&v^{(N)}_{tt} + \delta \omega _0 v^{(N)}_t + \omega _0^2 v^{(N)}  &   \\ =&\, - \mu p^{(N-1)}+\zeta (v^{(N-1)})^2 - \xi \left( 2 v^{(N-1)}v^{(N-1)}_{tt} + (v^{(N-1)}_t)^2 \right)  &   \text {in } \Omega \times (0,T),\\&v^{(N)}(0) = v^{(N)}(T), \ v^{(N)}_t(0) = v^{(N)}_t(T)  &   \text {in } \Omega . \end{aligned} \right. \end{aligned}$$

We proceed through several steps.

(i) Let $N=1$. Note that we can first solve (6b) and use its solution as the input for solving (6a). By Lemma 1, we have a unique $v^{(1)}\in \mathcal {X}_v$ that solves (6b), such that

$$\begin{aligned} \begin{aligned} \Vert v^{(1)}\Vert _{\mathcal {X}_v} \lesssim \Vert p^{(0)}\Vert _{L^2(L^\infty (\Omega ))}+ \Vert v^{(0)}\Vert ^2_{\mathcal {X}_v}. \end{aligned} \end{aligned}$$

From here, we find that

$$\begin{aligned} \begin{aligned} \Vert v^{(1)}\Vert _{\mathcal {X}_v} \lesssim r_p+ r_v^2, \end{aligned} \end{aligned}$$

and we can conclude that $\Vert v^{(1)}\Vert _{\mathcal {X}_v} \le r_v$ provided $r_p$ and $r_v$ are small enough. By Proposition 1, the linear problem in (6a) for $N=1$ has a unique solution $p^{(1)}\in \mathcal {X}_p$, such that

$$\begin{aligned} \begin{aligned} \Vert p^{(1)}\Vert _{\mathcal {X}_p} \lesssim&\, \Vert -\eta ((p^{(0)})^2)_{tt} - c^2 \rho _0 n_0 {v^{(1)}_{tt}} + h\Vert _{L^2(L^2(\Omega ))} \\ \lesssim&\, \Vert p^{(0)}\Vert ^2_{\mathcal {X}_p} + \Vert n_0\Vert _{L^\infty (\Omega )}\Vert v^{(1)}\Vert _{\mathcal {X}_v} + \Vert h\Vert _{L^2(L^2(\Omega ))}. \end{aligned} \end{aligned}$$

By using the fact that $\Vert v^{(1)}\Vert _{\mathcal {X}_v} \le r_v$, we obtain

$$\begin{aligned} \begin{aligned} \Vert p^{(1)}\Vert _{\mathcal {X}_p} \lesssim r_p^2 + \Vert n_0\Vert _{L^\infty (\Omega )} r_v+ \Vert h\Vert _{L^2(L^2(\Omega ))}. \end{aligned} \end{aligned}$$

From here, we can conclude that $\Vert p^{(1)}\Vert _{\mathcal {X}_p} \le r_p$ for small enough $\Vert h\Vert _{L^2(L^2(\Omega ))}$ by reducing $r_p$ and $\Vert n_0\Vert _{L^\infty (\Omega )}$.

(ii) Let now $(p^{(N-1)}, v^{(N-1)}) \in \mathbb {B}_{r_p, r_v}$. By Lemma 1, we have

$$\begin{aligned} \begin{aligned} \Vert v^{(N)}\Vert _{\mathcal {X}_v} \lesssim \Vert p^{(N-1)}\Vert _{L^2(L^\infty (\Omega ))}+ \Vert v^{(N-1)}\Vert ^2_{\mathcal {X}_v} \end{aligned} \end{aligned}$$

and by Proposition 1, the linear problem in (6a) with periodic time conditions has a unique solution $p^{(N)}\in \mathcal {X}_p$, such that

$$\begin{aligned} \begin{aligned} \Vert p^{(N)}\Vert _{\mathcal {X}_p} \le&\, C \Vert -\eta ((p^{(N-1)})^2)_{tt} - c^2 \rho _0 n_0 v^{(N)}_{tt} + h\Vert _{L^2(L^2(\Omega ))}\\ \lesssim&\, \Vert p^{(N-1)}\Vert ^2_{\mathcal {X}_p} + \Vert n_0\Vert _{L^\infty (\Omega )}\Vert v^{(N)}\Vert _{\mathcal {X}_v} + \Vert h\Vert _{L^2(L^2(\Omega ))}. \end{aligned} \end{aligned}$$

By then proceeding analogously to the case $N=1$, we obtain

$$\begin{aligned} \begin{aligned} \Vert p^{(N)}\Vert _{\mathcal {X}_p} \le r_p, \quad \Vert v^{(N)}\Vert _{\mathcal {X}_v} \le r_v, \end{aligned} \end{aligned}$$

provided $\Vert n_0\Vert _{L^\infty (\Omega )}$ and $\Vert h\Vert _{L^2(L^2(\Omega ))}$ are small enough. We thus conclude that for small enough $\Vert h\Vert _{L^2(L^2(\Omega ))}$ and $\Vert n_0\Vert _{L^\infty (\Omega )}$, there exist $r_p>0$ and $r_v>0$, such that if $(p^{(0)}, v^{(0)}) \in \mathbb {B}_{r_p, r_v}$, then for each $N \ge 1$, problem (6) has a unique solution $(p^{(N)}, v^{(N)}) \in \mathbb {B}_{r_p, r_v}$.

(iii) We next wish to show that $\{(p^{(N)}, v^{(N)})\}_{N \ge 1}$ is a Cauchy sequence in $\mathcal {X}_p\times \mathcal {X}_v$. We note that for $N_1$, $N_2 \in \mathbb {N}$, the difference $(\overline{p}, \overline{v})=(p^{(N_1)}-p^{(N_2)}, v^{(N_1)}-v^{(N_2)})$ solves

$$\begin{aligned} \left\{ \begin{aligned} \  &\overline{p}_{tt} - c^2 \Delta \overline{p}- b \Delta \overline{p}_t  &   \\ =&\, -\eta ((p^{(N_1-1)}-p^{(N_2-1)})(p^{(N_1-1)}+p^{(N_2-1)}))_{tt} - c^2 \rho _0 n_0 (v^{(N_1)}_{tt}-v^{(N_2)}_{tt}) \,  &   \text {in } \Omega ,\\&\beta \overline{p}_t + \gamma \overline{p}+ \nabla \overline{p}\cdot \boldsymbol{n}= 0  &   \text {on } \partial \Omega , \hspace{-0.28cm}\\ \end{aligned} \right. \end{aligned}$$

and From here, similarly to before, using Proposition 1 and Lemma 1, we obtain

8 $$\begin{aligned} \begin{aligned} \Vert p^{(N_1)}-p^{(N_2)}\Vert _{\mathcal {X}_p} \lesssim r_p\Vert p^{(N_1-1)}-p^{(N_2-1)}\Vert _{\mathcal {X}_p} + \Vert n_0\Vert _{L^\infty (\Omega )}\Vert v^{(N_1)}-v^{(N_2)}\Vert _{\mathcal {X}_v} \end{aligned} \end{aligned}$$

and9By adding $(8)+ \lambda \cdot (9)$, with $\lambda >0$, we obtain

$$\begin{aligned} \begin{aligned}&\Vert p^{(N_1)}-p^{(N_2)}\Vert _{\mathcal {X}_p} + (\lambda - C \Vert n_0\Vert _{L^\infty (\Omega )})\Vert v^{(N_1)}-v^{(N_2)}\Vert _{\mathcal {X}_v}\\ \le&\, C ( \lambda + r_p) \Vert p^{(N_1-1)}-p^{(N_2-1)}\Vert _{\mathcal {X}_p} + C \lambda r_v\Vert v^{(N_1-1)}-v^{(N_2-1)}\Vert _{\mathcal {X}_v} \end{aligned} \end{aligned}$$

for some $C>0$. We then impose $C \Vert n_0\Vert _{L^\infty (\Omega )} < \lambda$, and choose $r_p$, $\lambda$, and $r_v$ sufficiently small to conclude that $\{(p^{(N)}, v^{(N)})\}_{N \ge 1}$ is a Cauchy sequence. Thus, there exists (*p*, *v*) such that $\{(p^{(N)}, v^{(N)})\}_{N \ge 1}$ converges to it in $\mathcal {X}_p\times \mathcal {X}_v$. Passing to the limit in (6) proves that (*p*, *v*) solves the pressure-volume problem.

(iv) Finally, to show that such a constructed solution is unique, we take

$$ (p^{<1>}, v^{<1>}), \, (p^{<2>}, v^{<2>}) \in \mathbb {B}_{r_p, r_v}, $$

and note that the difference $(\overline{p}, \overline{v})=(p^{<1>}-p^{<2>}, v^{<1>}-v^{<2>})$ solves

$$\begin{aligned} \left\{ \begin{aligned} \  &\overline{p}_{tt} - c^2 \Delta \overline{p}- b \Delta \overline{p}_t = -\eta (\overline{p}(p^{<1>}+p^{<2>}))_{tt} - c^2 \rho _0 n_0 \overline{v}_{tt} \,  &   \text {in } \Omega \times (0,T),\\&\beta \overline{p}_t + \gamma \overline{p}+ \nabla \overline{p}\cdot \boldsymbol{n}= 0  &   \text {on } \partial \Omega \times (0,T), \hspace{-0.28cm}\\ \end{aligned} \right. \end{aligned}$$

and

$$\begin{aligned} \begin{aligned}&\overline{v}_{tt} + \delta \omega _0 \overline{v}_t + \omega _0^2 \overline{v}=- \mu \overline{p}+ \zeta \overline{v}\left( v^{<1>}+v^{<2>}\right) \\&\quad - \xi \left( 2 (\overline{v}v^{<1>}_{tt}+v^{<2>}\overline{v}_{tt} ) + \overline{v}_t(v^{<1>}_t+v^{<2>}_t)\right) . \end{aligned} \end{aligned}$$

We obtain analogously to before

$$\begin{aligned} \begin{aligned} \Vert \overline{p}\Vert _{\mathcal {X}_p} \lesssim r_p\Vert \overline{p}\Vert _{\mathcal {X}_p}+ \Vert n_0\Vert _{L^\infty (\Omega )} \Vert \overline{v}\Vert _{\mathcal {X}_v} \end{aligned} \end{aligned}$$

and

$$\begin{aligned} \begin{aligned} \Vert \overline{v}\Vert _{\mathcal {X}_v} \lesssim \Vert \overline{p}\Vert _{\mathcal {X}_p}+ r_v\Vert \overline{v}\Vert _{\mathcal {X}_v}. \end{aligned} \end{aligned}$$

For $r_v$, $r_p$, and $\Vert n_0\Vert _{L^\infty (\Omega )}$ small enough, these inequalities allow us to conclude that $\Vert \overline{p}\Vert _{\mathcal {X}_p}=\Vert \overline{v}\Vert _{\mathcal {X}_v}=0$. This step completes the proof. $\square$

The small-solution setting of Theorem 1 effectively forces the nonlinear ODE to retain the damped harmonic oscillator structure with *T*-periodic coefficients. Indeed, under the assumptions of Theorem 1, for $r_v$ small enough, so that

$$\begin{aligned} \max \, \left\{ 2| \xi |, \frac{1}{\delta \omega _0}| \xi |, \frac{1}{\omega _0^2} |\zeta | \right\} \cdot r_v<1, \end{aligned}$$

we can rewrite the ODE in the following form:

$$\begin{aligned} \begin{aligned} v_{tt}+ \frac{\delta \omega _0-\xi v_t}{1-2 \xi v} v_t+ \frac{\omega _0^2-\zeta v}{1-2 \xi v} v =- \frac{\mu }{1-2\xi v} p. \end{aligned} \end{aligned}$$

Setting $\ell = \dfrac{\delta \omega _0-\xi v_t}{1-2 \xi v}>0$, $q =\dfrac{\omega _0^2-\zeta v}{1-2 \xi v}>0$, and $f = - \dfrac{\mu }{1-2\xi v} p$, we see that the volume fluctuation *v* can be represented as the solution of a second-order ODE (pointwise a.e. in space) with positive *T*-periodic coefficients and a *T*-periodic right-hand side, namely

$$\begin{aligned} \begin{aligned}&v_{tt}+\ell (t)v_t+q(t) v =f(t), \end{aligned} \end{aligned}$$

where $$\ell (0)=\ell (T)$$, $$q(0)=q(T)$$, $$f(0)=f(T)$$.

### On the Smallness Assumption

The assumption (5) on the smallness of the microbubble number density $n_0$ in Theorem 1 regulates the strength of the source of acoustic waves, along with the smallness of the external source *h*. To assess how meaningful this assumption is, we consider a nondimensional system obtained by introducing the scaling

$$\begin{aligned} \begin{aligned} x = L \tilde{x}, \qquad t = \frac{L}{c} \tilde{t}, \quad p = p^{ref }\, \tilde{p}, \qquad v = v^{ref }\, \tilde{v}, \end{aligned} \end{aligned}$$

where *L* is a characteristic length scale, $p^{ref }$ is a reference pressure amplitude, and $v^{ref }$ a reference volume. After the change of variables and division by $\frac{c^2 p^{ref }}{L^2}$, we obtain the dimensionless pressure equation:

$$\begin{aligned} \begin{aligned}&\ \ \tilde{p}_{tt} - \Delta \tilde{p} - \tilde{b} \Delta \tilde{p}_t = \tilde{\eta } (\tilde{p}^2)_{tt} + \tilde{\kappa } {n}_0 \tilde{v}_{tt} +\tilde{ \alpha } h \quad  &   \text {in } \Omega \times (0,T), \end{aligned} \end{aligned}$$

where the relevant transformed coefficient is given by

$$\begin{aligned} \tilde{\kappa } {n}_0 = \frac{ \rho _0 c^2 v^{ref }}{p^{ref }} {n}_0. \end{aligned}$$

Since $v^{ref }n_0$ can be understood as the gas volume fraction (see [33, Sec. 2]), the smallness assumption corresponds to requiring moderate concentrations of microbubbles. For typical parameters in medical imaging, $c \sim 1500\hbox {m/s}$, $\rho \sim 1000\, \hbox {kg/m}^3$, $p^{ref }\in [10^5, 10^6]\,$Pa, and $R_0 \sim 10^{-6}\,$m, $v^{ref }= \frac{4}{3} R_0^3 \pi$, we obtain $\tilde{\kappa } n_0 \ll 1$ for microbubble densities in the range $n_0 \in [10^9, 10^{12}]\,$ bubbles$/m^3$.

## Time Discretization Via a Multiharmonic Ansatz for Real Fields

In this section, we discuss the time discretization of the system by using a multiharmonic Ansatz, in the general spirit of [21]. We focus on a special case of having *T*-periodic acoustic excitation. That is, we assume that the acoustic source term has the form $h=g_{tt}$, where the function *g* is given by

11 $$\begin{aligned} g(x,t)=\mathfrak {R}\left\{ \sum _{m=0}^M \exp (\imath m \omega t)h_m^M(x)\right\} , \quad \omega = \dfrac{2 \pi }{T}, \quad h_m^M \in L^2(\Omega ; \mathbb {C}). \end{aligned}$$

Under the assumptions of Theorem 1, a unique time-periodic solution (*p*, *v*) of the system given in (3) exists. For numerical approximations, we thus employ the following multiharmonic Ansatz:

12 $$\begin{aligned} \begin{aligned} u^N(x,t)=&\, \frac{1}{2} \sum _{m=0}^{N} \left( \exp (\imath m \omega t) u_m^N(x) + \exp (-\imath m \omega t) \overline{u^N_m(x)} \right) \\ =&\, \mathfrak {R}\left\{ \sum _{m=0}^{N} \exp (\imath m \omega t) u_m^N(x)\right\} . \end{aligned} \end{aligned}$$

Alternatively, the multiharmonic Ansatz can be written in a more standard Fourier series form:

$$\begin{aligned} u^N(x,t) = \sum _{m=0}^N \left[ u^c_m(x) \cos (m \omega t)+ u^s_m(x)\sin (m\omega t)\right] \end{aligned}$$

with $u^N_m(x) = u_m^c(x)- \imath u_m^s(x)$. We then look for the approximate pressure-volume field $(p^N, v^N) \in X_N\times X_N$, with

$$\begin{aligned} X_N = \left\{ \mathfrak {R}\left\{ \sum _{m=0}^N \exp ( \imath m \omega t) u_m^N(x) \right\} \, : \, u_m^N \in H^2(\Omega ; \mathbb {C}) \right\} , \end{aligned}$$

where the coefficients $(p^N_m, v^N_m)$ in the expansion are determined from the following system:

13 $$\begin{aligned} \left\{ \begin{aligned} \  &p_{tt}^N- c^2 \Delta p^N- b \Delta p_t^N  &   \\ =&\, \eta \,Proj _{X_N}\left[ ((p^N)^2)_{tt}\right] +c^2 \rho _0 n_0 v^N+ Proj _{X_N}h  &   \text {in } \Omega \times (0,T),\\&\beta p_t^N+ \gamma p^N+ \nabla p^N\cdot \boldsymbol{n}= 0  &   \ \text {on } \partial \Omega \times (0,T),\\&v_{tt}^N+ \delta \omega _0 v_t^N+ \omega _0^2 v^N  &   \\ =&\, - \mu p^N+ \,Proj _{X_N}\left[ \zeta (v^N)^2 + \xi \left( 2 v^Nv_{tt}^N+ (v_t^N)^2 \right) \right]  &   \text {in } \Omega \times (0,T). \end{aligned} \right. \end{aligned}$$

We next show that (13) can be equivalently rewritten as a system of Helmholtz problems and algebraic equations for computing $(p^N_m, v^N_m)$.

### A Multiharmonic Cut-Off Algorithm

Below we skip writing the dependencies of Fourier coefficients on *x* for readability.

#### Proposition 2

Let the assumptions of Theorem 1 hold with the acoustic source term assumed to have the form $h=g_{tt}$, where *g* is given in (11). The problem in (13) yields the following coupled system for computing $(p^N_m, v^N_m)$ for $N \ge 0$ and $0 \le m \le N$:

$$\begin{aligned} m = 0:&{\left\{ \begin{array}{ll} \begin{aligned} \text {(i)} &  \quad p_0^N =0, \\ \text {(ii)} &  \quad \omega _0^2 v_0^N = \zeta \left( v_0^N \right) ^2 + \sum _{j=1}^N \left( \frac{\zeta }{2} - \frac{\xi }{2} \omega ^2 j^2 \right) \left| v_j^N \right| ^2, \\ \end{aligned} \end{array}\right. } \\ m= 1:&{\left\{ \begin{array}{ll} \begin{aligned} \text {(i)} &  \quad - p_1^N - \frac{c^2 + \imath b \omega }{\omega ^2} \Delta p_1^N+ c^2 n_0\rho _0 v_1^N = - h_1^N - \sum _{k=3:2}^{2N-1} \eta \overline{p_{\frac{k-1}{2}}^N} p_{\frac{k+1}{2}}^N, \\ \text {(ii)} &  \quad \left( - \omega ^2 + \imath \delta \omega _0 \omega + \omega _0^2 \right) v_1^N + \mu p_1^N\\ &  \quad = (\zeta -\xi \omega ^2) v_0^N v_1^N + \sum _{k=1:2}^{2N-1} \left( \zeta - \xi \omega ^2 \frac{k^2 + 3}{4}\right) \overline{v_{\frac{k-1}{2}}^N} v_{\frac{k+1}{2}}^N, \\ \end{aligned} \end{array}\right. } \\ m = \left\{ 2, \ldots , N \right\} :&{\left\{ \begin{array}{ll} \begin{aligned} \text {(i)} &  \quad - p_m^N- \frac{ c^2 + \imath m b \omega }{m^2 \omega ^2} \Delta p_m^N + c^2 \rho _0 n_0 v_m^N\\ &  \quad = - h_m^N -\eta \left( \sum _{l=1}^{m-1} p_l^N p_{m-l}^N- 2 \sum _{k=m+2:2}^{2N-m} \overline{p_{\frac{k-m}{2}}^N} p_{\frac{k+m}{2}}^N \right) , \\ \text {(ii)}&  \quad \left( - \omega ^2m^2 + \imath \delta \omega _0 \omega m+\omega _0^2\right) v_m^N + \mu p_m^N, \\ &  \quad = \sum _{l=0}^m \left( \frac{\zeta }{2} - \frac{\xi \omega ^2}{2} (m-l)(2m-l) \right) v_l^N v_{m-l}^N \\ &  \quad \quad + \sum _{k=m:2}^{2N-m} \left( \zeta - \xi \omega ^2 \frac{k^2 + 3m^2}{4}\right) \overline{v_{\frac{k-m}{2}}^N} v_{\frac{k+m}{2}}^N. \\ \end{aligned} \end{array}\right. } \end{aligned}$$

Additionally, each of the individual functions $p_m^N$ for $m \in \left\{ 1, \ldots , N \right\}$ satisfies the following boundary conditions:

14 $$\begin{aligned} (\imath \omega m \beta + \gamma ) p_m^N + \nabla p_m^N \cdot \boldsymbol{n}= 0 \qquad \text { on }\, \partial \Omega . \end{aligned}$$

#### Proof

We begin by considering the derivation of the multiharmonic algorithm for the Westervelt’s equation and the ODE separately. For the PDE, one can follow the steps outlined in [21], where the de-coupled Westervelt equation is considered, by setting the right-hand side to $g = h + c^2 \rho _0 n_0 v$. Projections onto $X_N$ of product terms appearing on the right-hand side of (13) can be expressed using [21, Lemma 3.1].

For the ODE in (13), the Ansatz given in (12), together with [21, Lemma 3.1], yields

$$\begin{aligned} 0 =&\mathfrak {R}\left\{ - \omega ^2 \sum _{m=0}^N v_m^N m^2 \exp (\imath m \omega t) + \delta \omega _0 \imath \omega \sum _{m=0}^N v_m^Nm \exp ( \imath m \omega t) + \omega _0^2 \sum _{m=0}^N v_m^N \exp (\imath m \omega t) \right. \\&\left. + \mu \sum _{m=0}^N p_m^N \exp (\imath m \omega t)- \frac{\zeta }{2} \left( (v_0^N)^2 + \sum _{j=0}^N \left| v_j^N\right| ^2 \right. \right. \\&\left. \left. + \sum _{m=1}^N \left[ \sum _{l=0}^m v_l^N v_{m-l}^N + 2 \sum _{k=m:2}^{2N-m} \overline{v_{\frac{k-m}{2}}^N} v_{\frac{k+m}{2}}^N \right] \exp (\imath m \omega t) \right) + \xi \frac{\omega ^2}{2}\left( \sum _{j=0}^N j^2 \left| v_j^N\right| ^2 \right. \right. \\&\left. \left. + \sum _{m=1}^N \left[ \sum _{l=0}^m (m-l)(2m-l) v_l^N v_{m-l}^N + \sum _{k=m:2}^{2N-m} \frac{k^2 +3m^2}{2} \overline{v_{\frac{k-m}{2}}^N} v_{\frac{k+m}{2}}^N \right] \exp (\imath m \omega t) \right) \right\} . \end{aligned}$$

From here, we have

$$\begin{aligned} 0 =&\mathfrak {R}\left\{ \sum _{m=0}^N \left[ \left( - \omega ^2 m^2 + \delta \omega _0 \imath \omega m + \omega _0^2 \right) v_m^N + \mu p_m^N \right] \exp (\imath m \omega t) \right. \\&\left. - \frac{\zeta }{2} (v_0^N)^2 + \sum _{j=0}^N \left( - \frac{\zeta }{2} + \xi \frac{\omega ^2}{2} j^2 \right) \left| v_j^N\right| ^2 + \sum _{m=1}^N \left[ \sum _{l=0}^m \left( -\frac{\zeta }{2} + \xi \frac{\omega ^2}{2} (m-l)(2m-l) \right) v_l^N v_{m-l}^N \right. \right. \\&\left. \left. + \sum _{k=m:2}^{2N-m} \left( -\zeta + \xi \omega ^2 \frac{k^2 + 3m^2}{4}\right) \overline{v_{\frac{k-m}{2}}^N} v_{\frac{k+m}{2}}^N \right] \exp (\imath m \omega t) \right\} . \end{aligned}$$

Combining our derivations above and using linear independence of the functions $t \mapsto \exp ( \imath m \omega t)$ yields a coupled nonlinear system for the functions $\left\{ p_0^N, v_0^N, \cdots , p_N^N, v_N^N \right\}$. Additionally, each of the individual functions $p_m^N$ must satisfy the boundary conditions in (14). The 0-th equation is given by $\Delta p^N_0 = 0$, which together with the 0-th boundary condition (and the fact that $\gamma >0$) implies that $p_0^N$ vanishes. By dividing the resulting PDEs by $m^2\omega ^2$ for $m \ge 1$, we arrive at the claimed coupled system. $\square$

We observe that without the sums over *k* on the right-hand side of the system in Proposition 2, the system would be triangular and could be solved by substitution. One way of obtaining a triangular form is by linearizing the right-hand side as we show next.

### A Linearized Multiharmonic Cut-Off Algorithm

Next, we propose a linearized multilevel method. That is, for $N \ge 1$, we consider the sequence of the following linearized equations where the quadratic terms are approximated by taking into account $N-1$ harmonics:

15 $$\begin{aligned} \left\{ \begin{aligned} \  &p_{tt}^N- c^2 \Delta p^N- b \Delta p_t^N  &   \\ =&\, \eta \,Proj _{X_N}\left[ ((p^{N-1})^2)_{tt}\right] +c^2 \rho _0 n_0 v_{tt}^N+ Proj _{X_N}h  &   \text {in } \Omega \times (0,T),\\&\beta p_t^N+ \gamma p^N+ \nabla p^N\cdot \boldsymbol{n}= 0  &   \text {on } \partial \Omega \times (0,T),\\&v_{tt}^N+ \delta \omega _0 v_t^N+ \omega _0^2 v^N  &   \\ =&\, - \mu p^N+ \,Proj _{X_N}\left[ \zeta (v^{N-1})^2 + \xi \left( 2 v^{N-1}v_{tt}^{N-1}+ (v_t^{N-1})^2 \right) \right]  &   \text {in } \Omega \times (0,T), \end{aligned} \right. \end{aligned}$$

where for $u^N \in X_N$, we formally define $u_m^N = 0$ for all $m>N$. To start the algorithm, we set $p^0 =0$ and $v^0 = 0$. We next show that this problem can be seen as a triangular system of Helmholtz problems and algebraic equations that can be solved by successive substitution.

#### Proposition 3

Let the assumptions of Theorem 1 hold with the acoustic source term assumed to have the form $h=g_{tt}$, where *g* is given in (11). Then, an equivalent formulation of the problem in (15) with $p^0=v^0=0$ is given by the following coupled system:

$$\begin{aligned} m = 0:&{\left\{ \begin{array}{ll} \begin{aligned} \text {(i)} &  \quad p_0^N =0, \\ \text {(ii)} &  \quad \omega _0^2 v_0^N= \frac{\zeta }{2} \left( v_0^{N-1} \right) ^2 + \sum _{j=0}^{N-1} \left( \frac{\zeta }{2} - \frac{\xi \omega ^2}{2}j^2 \right) \left| v_j^{N-1} \right| ^2,\\ \end{aligned} \end{array}\right. } \\ m= 1:&{\left\{ \begin{array}{ll} \begin{aligned} \text {(i)} &  \quad - p_1^N - \frac{ c^2 + \imath b \omega }{\omega ^2} \Delta p_1^N+ c^2 \rho _0 n_0 v_1^{N} = -h_1^N - \eta \sum _{k=3:2}^{2N-3} \overline{p_{\frac{k-1}{2}}^{N-1}} p_{\frac{k+1}{2}}^{N-1}, \\ \text {(ii)} &  \quad \frac{1}{\alpha _1}v_1^N + \mu p_1^{N} = \left( \zeta - \xi \omega ^2\right) v_0^{N-1}v_1^{N-1} \\ &  \hspace{2.5cm} + \sum _{k=1:2}^{2N-3} \left( \zeta - \xi \omega ^2 \frac{k^2 + 3}{4}\right) \overline{v_{\frac{k-1}{2}}^{N-1}} v_{\frac{k+1}{2}}^{N-1}, \\ \end{aligned} \end{array}\right. } \\ m = \left\{ 2, \ldots , N \right\} :&{\left\{ \begin{array}{ll} \begin{aligned} \text {(i)} &  \quad - p_m^N- \frac{\left( c^2 + \imath m b \omega \right) }{ \omega ^2 m^2}\Delta p_m^N + c^2 \rho _0 n_0 v_m^{N}\\ &  \quad = - h_m^N - \frac{ \eta }{2} \left( \sum _{l=1}^{m-1} p_l^{N-1} p_{m-l}^{N-1} -2 \sum _{k = m+2:2}^{2(N-1)-m} \overline{p_{\frac{k-m}{2}}^{N-1}} p_{\frac{k+m}{2}}^{N-1} \right) , \\ \text {(ii)}&  \quad \frac{1}{\alpha _m} v_m^N + \mu p_m^{N} = \sum _{l=0}^m \left( \frac{\zeta }{2} - \frac{\xi \omega ^2}{2} (m-l)(2m-l) \right) v_l^{N-1} v_{m-l}^{N-1} \\ &  \hspace{2.5cm} + \sum _{k=m:2}^{2(N-1)-m} \left( \zeta - \frac{\xi \omega ^2 (k^2 + 3 m^2)}{4} \right) \overline{v_{\frac{k-m}{2}}^{N-1}} v_{\frac{k+m}{2}}^{N-1} \end{aligned} \end{array}\right. } \end{aligned}$$

for $N \ge 1$, where for $m \ge 1$, $\alpha _m = \left( - m^2\omega ^2 + \imath m \delta \omega _0 \omega +\omega _0^2\right) ^{-1}$. Additionally, each of the individual functions $p_m^N$, where $m \in \left\{ 1, \ldots , N \right\}$, should satisfy boundary conditions (14).

#### Proof

The statement follows analogously to before by applying the Ansatz given in (12) and making use of the expressions provided in [21, Lemma 3.1] for projections onto $X_N$ of products of two functions in $X_N$. $\square$

Looking at the system in Proposition 3, we see that we can express the volume harmonics as

16 $$\begin{aligned} v^N_m = -\alpha _m \mu p^N_m + \alpha _m f_v^{N-1} \text { for } \ m \ge 1, \end{aligned}$$

where

$$\begin{aligned} f^{N-1}_v&= \sum _{l=0}^m \left( \frac{\zeta }{2} - \frac{\xi \omega ^2}{2} (m-l)(2m-l) \right) v_l^{N-1} v_{m-l}^{N-1}\\&\quad + \sum _{k=m:2}^{2(N-1)-m} \left( \zeta - \frac{\xi \omega ^2 (k^2 + 3 m^2)}{4} \right) \overline{v_{\frac{k-m}{2}}^{N-1}} v_{\frac{k+m}{2}}^{N-1}. \end{aligned}$$

By substituting these values into the equations for $p^N_m$, the coupled system in Proposition 3 can be rewritten as a system of (16) and Helmholtz equations given by

17 $$\begin{aligned} \left\{ \begin{aligned}&-\left( 1+\imath \omega \frac{m b}{c^2} \right) \Delta p^N_m - (k^2+\mathfrak {a}) p^N_m +\imath \mathfrak {b}p^N_m = -k^2 h_m^N - f_{p,v}^{N-1} \quad \text { in }\, \Omega ,\quad \text {with } k= \frac{m \omega }{c},\\&(\imath \omega m \beta + \gamma ) p^N_m + \nabla p^N_m \cdot \boldsymbol{n}= 0 \quad \text { on }\, \partial \Omega , \end{aligned} \right. \end{aligned}$$

where the source is

$$\begin{aligned} f_{p,v}^{N-1} = \alpha _m \omega ^2 m^2 \rho _0 n_0 f^{N-1}_v + k^2 \frac{ \eta }{2} f^{N-1}_p \end{aligned}$$

with

$$ f^{N-1}_p = \sum _{l=1}^{m-1} p_l^{N-1} p_{m-l}^{N-1} -2 \sum _{k = m+2:2}^{2(N-1)-m} \overline{p_{\frac{k-m}{2}}^{N-1}} p_{\frac{k+m}{2}}^{N-1}. $$

In (17), we have

$$\begin{aligned} \mathfrak {a}= \mu \rho _0 n_0 \frac{m^2 \omega ^2 (\omega _0^2 - m^2 \omega ^2)}{(\omega _0^2-m^2\omega ^2)^2 + (m \delta \omega _0 \omega )^2}\ \text { and } \ \mathfrak {b}= \mu \rho _0 n_0 \frac{m^3 \omega ^3 \delta \omega _0 }{(\omega _0^2-m^2\omega ^2)^2 + (m \delta \omega _0 \omega )^2}. \end{aligned}$$

Note that $\mathfrak {b}>0$, while the sign of $\mathfrak {a}$ depends on the relation between $\omega _0$ and $m \omega$. The form of the Helmholtz equation in (17) reveals more clearly the influence of microbubbles on the wave propagation, through the dissipation signaled by the $i \mathfrak {b}p^N_m$ term and modification of the wave number via the $-(k^2+\mathfrak {a}) p^N_m$ term. $\square$

## Convergence of the Linearized Multiharmonic Algorithm for Real Fields

We next wish to establish convergence of the iterative scheme in (15) as $N \rightarrow \infty$ in a suitable norm. We can mimic the proof of Theorem 1 to show by induction that for each $N \ge 1$, problem (15) has a unique solution $(p^N, v^N) \in X_N\times X_N$. For fixed $N \ge 1$, the existence and uniqueness of $p^N$ follow by noting that, thanks to Proposition 3, harmonics $p^N_m$ are obtained as solutions of the Helmholtz problems in (17). Furthermore, an analogous testing procedure to the one in Theorem 1 applied on (15) yields

$$ \Vert p^N\Vert _{\mathcal {X}_p} \le r_p, \quad \Vert v^N\Vert _{\mathcal {X}_v} \le r_v, $$

provided $r_p$, $r_v$, and $\Vert \mu \Vert _{L^\infty (\Omega )}$ are sufficiently small. We omit these details here and focus on establishing convergence. The convergence will be shown in the norms of the spaces $Y^0_p$ and $Y^0_{v}$, where

$$\begin{aligned} \begin{aligned} Y_p^\ell =&\, H^{\ell +1}(0,T; H^1(\Omega )) \cap H^{\ell +2}(0,T; L^2(\Omega )) \cap H^{\ell +2}(0,T; L^2(\partial \Omega )),\\ Y_{v}^\ell =&\, H^{\ell +2}(0,T; L^2(\Omega )) \end{aligned} \end{aligned}$$

for $\ell \ge 0$. To make writing more compact, we introduce the short-hand norm notation

$$\begin{aligned} |\!|\!|(p,v)|\!|\!|_{Y^\ell _p\times Y^\ell _{v}} :=\Vert p\Vert _{Y^\ell _p}+\Vert v\Vert _{Y^\ell _{v}} \end{aligned}$$

for $(p,v) \in Y^\ell _p\times Y^\ell _{v}$, and we also adopt the operator notationand

$$\begin{aligned} <\mathcal {L}_2 v^N, \phi ^N> :=\int _0^T \int _{\Omega }\left( v_{tt}^N+ \delta \omega _0 v_t^N+ \omega _0^2 v^N\right) \phi ^N\, d xd s, \quad \phi ^N\in X_N. \end{aligned}$$

Then the algorithm can be restated as follows. Given the previous iterate $(p^{N-1}, v^{N-1}) \in X_N\times X_N$, we compute $(p^N, v^N)$ for $N \ge 1$ as the solution of

$$\begin{aligned} \begin{aligned} <\mathcal {L}_1 p^N, \phi ^N> =&\, \int _0^T \int _{\Omega }\left( {\eta } ((p^{N-1})^2)_{tt} + c^2 \rho _0 n_0 v_{tt}^N+h\right) \phi ^N\, d xd s, \end{aligned} \end{aligned}$$

and for all $\phi ^N\in X_N$.

In the convergence proof, we will involve the truncated Fourier series of a continuous-in-time function *u* given by

$$\begin{aligned} \begin{aligned} \tilde{u}^N(x,t)= \sum _{m=0}^N \left[ u^{c}_m (x) \cos (m \omega t)+ u_m^{s}(x)\sin (m \omega t)\right] \in X_N, \quad N \ge 0, \end{aligned} \end{aligned}$$

with the coefficients

$$\begin{aligned} \begin{aligned} u_m^{c}(x) = \frac{2}{T} \int _0^T u(x,t) \cos (m \omega t) \, d t, \quad u_m^{s}(x) = \frac{2}{T} \int _0^T u(x,t) \sin (m \omega t) \, d t. \end{aligned} \end{aligned}$$

By integrating the time-periodic Westervelt equation and acoustic boundary conditions in (3) over (0, *T*), we can conclude that $\int _0^T p(x,t) \, d t=0$ in $\Omega$. Thus $\tilde{p}^0=0$, which is reflected by having $p^N_0=0$ in the multiharmonic algorithms.

It can be shown analogously to [3, Lemma 12] that the following error bound holds for $\ell \ge 1$:

19 $$\begin{aligned} \begin{aligned} |\!|\!|(p-\tilde{p}^N, v-\tilde{v}^N)|\!|\!|_{(Y^0_p\cap H^2(H^1(\Omega ))) \times Y^0_{v}} \le&\, C N^{-\ell } (\Vert p\Vert _{Y_p^{\ell } \cap H^{\ell +2}(H^1(\Omega ))}+\Vert v\Vert _{Y_v^{\ell }}), \end{aligned} \end{aligned}$$

which we can exploit to characterize the error of the scheme in the next statement. We include the proof of (19) in Appendix B for completeness.

### Theorem 2

Let the assumptions of Theorem 1 hold with the acoustic source term $h=g_{tt}$, where *g* is given in (11). Assume additionally that

$$\begin{aligned} \begin{aligned} (p,v) \in \left( \mathcal {X}_p\cap Y_p^\ell \cap H^{\ell +2}(0,T; H^1(\Omega )) \right) \times \left( \mathcal {X}_v\cap Y_{v}^\ell \right) , \quad \ell \ge 1. \end{aligned} \end{aligned}$$

Let $p^0=v^0=0$ and assume that $(p^N, v^N)$ are computed using (18) for $N \ge 1$. Then, provided $r_p$, $r_v$, and $\Vert \mu \Vert _{L^\infty (\Omega )}$ are sufficiently small, there exists $q=q(r_p, r_v)<1$, such that20where $C>0$ does not depend on *N*.

### Proof

The proof follows by splitting the error as follows:

$$\begin{aligned} \begin{aligned} p -p^N=&\, (p- \tilde{p}^N)- (p^N- \tilde{p}^N) {=}{:}err (\tilde{p}^N)-e^{p,N}, \\ v -v^N=&\, (v-\tilde{v}^N) -(v^N-\tilde{v}^N) {=}{:}err (\tilde{v}^N)-e^{v,N}, \end{aligned} \end{aligned}$$

and then representing $(e^{p,N}, e^{v,N})$ as the solution of a suitable semi-discrete wave-volume system. The discrete error $(e^{p,N}, e^{v,N})$ can be seen as the solution of 21aand21b for all $\phi ^N\in X_N$. Note that in the testing procedure for this problem we can exploit the fact that $\int _0^T \frac{d }{d t}(\cdot ) \, d t=0$ for time-periodic functions. By testing (21a) with $e^{p,N}$, $e_t^{p,N}$, and $e_{tt}^{p,N}$, employing Young’s and Hölder’s inequalities, and combining the resulting estimates, we can derive the following bound:22where we have also exploited the equivalence of the norms $\Vert w\Vert _{H^1(\Omega )}$ and $\Vert \nabla w\Vert _{L^2(\Omega )}+\Vert w\Vert _{L^2(\partial \Omega )}$ (see [32, Theorem 1.9]). The derivation of (22) is provided in Appendix C.

Then by using the rewriting

$$\begin{aligned} \begin{aligned} ((p^{N-1})^2)_{tt}-(p^2)_{tt}&=\, 2(p^{N-1}_t-p_t)(p^{N-1}_t+p_t)+2(p^{N-1}-p)p^{N-1}_{tt}\\&\quad +2 p(p^{N-1}_{tt}-p_{tt}), \end{aligned} \end{aligned}$$

and the fact that $\Vert p\Vert _{\mathcal {X}_p}$, $\Vert p^N\Vert \le r_p$ for all $N \ge 1$, we obtainFrom here via $p-p^N=err (\tilde{p}^N)-e^{p,N}$ and the triangle inequality, we arrive at23Similarly, testing (21b) with $e^{v,N}$, $e_t^{v,N}$, and $e_{tt}^{v,N}$ yields, after standard manipulations,By using the fact that $\Vert v\Vert _{\mathcal {X}_v}$, $\Vert v^N\Vert _{\mathcal {X}_v} \le r_v$ for all $N \ge 1$, and the triangle inequality, from (21b) we then have24Adding $\lambda \cdot$(24) and (23) with $\lambda >0$ small enough, so that the term $\lambda \Vert p^N-p\Vert _{L^2(L^2(\Omega ))}$ can be absorbed by the left-hand side, and then possibly reducing $\Vert n_0\Vert _{L^\infty (\Omega )}$ so that the term $\Vert n_0\Vert _{L^\infty (\Omega )} \Vert v_{tt}^N-v_{tt}\Vert _{L^2(L^2(\Omega ))}$ can be absorbed, yieldsBy possibly further reducing $r_p$ and $r_v$, we obtain $q(r_p, r_v) <1$, such thatfor some $C_0>0$. The statement then follows by employing (19). $\square$

Convergence of the scheme then follows in a straightforward manner from (22).

### Corollary 1

(Convergence of the linearized scheme) Let the assumptions of Theorem 2 hold. Then the solution $(p^N, v^N) \in X_N\times X_N$ of the iteration scheme (18) converges with respect to the norm $|\!|\!|(\cdot , \cdot )|\!|\!|_{Y^0_p\times Y^0_{v}}$ to the solution $(p, v) \in \mathbb {B}_{r_p, r_v}$ of (3) as $N \rightarrow \infty$.

### Proof

From (20), by iteration we obtainfor some $C>0$, independent of *N*. The statement then follows analogously to [34, Theorem 3.1], where the multiharmonic discretization of the de-coupled Westervelt equation is studied. We thus omit the details here. $\square$

### Setting the Zeroth Microbubble Harmonic to Zero

Unlike with the Westervelt equation, from the equation for *v*, we cannot in general conclude by integrating from 0 to *T* that $\int _0^T v(t) \, d t=0$. Nevertheless, in numerical methods for solving multiharmonic problems it is often assumed that the zeroth harmonic does not contribute significantly to the dynamics; this assumption is also made in the formal two-harmonic generation study for the linearized wave-ODE model with $b=\eta =0$ in [17, Ch. 5.3.2].

With the assumption $p_0^N = v_0^N = 0$ for all $N \ge 0$, the system derived in Proposition 3 further simplifies to

25 $$\begin{aligned} m= 1:&{\left\{ \begin{array}{ll} \begin{aligned} \text {(i)} &  \quad - p_1^{N} - \frac{ c^2 + \imath b \omega }{\omega ^2} \Delta p_1^N+ c^2 \rho _0 n_0 v_1^{N} = -h_1^N - \sum _{k=3:2}^{2N-3} \eta \overline{p_{\frac{k-1}{2}}^{N-1}} p_{\frac{k+1}{2}}^{N-1} \\ \text {(ii)} &  \quad v_1^N = \alpha _1 \left( - \mu p_1^{N} + \sum _{k=3:2}^{2N-3} \left( \zeta - \xi \omega ^2 \frac{k^2 + 3}{4}\right) \overline{v_{\frac{k-1}{2}}^{N-1}} v_{\frac{k+1}{2}}^{N-1}\right) \\ \end{aligned} \end{array}\right. } \end{aligned}$$

$$\begin{aligned} m = \left\{ 2, \ldots , N \right\} :&{\left\{ \begin{array}{ll} \begin{aligned} \text {(i)} &  \quad - p_m^N- \frac{\left( c^2 + \imath m b \omega \right) }{ \omega ^2 m^2}\Delta p_m^N + c^2 \rho _0 n_0 v_m^{N}\\ &  \quad = - h_m^N - \frac{ \eta }{2} \left( \sum _{l=1}^{m-1} p_l^{N-1} p_{m-l}^{N-1} -2 \sum _{k = m+2:2}^{2(N-1)-m} \overline{p_{\frac{k-m}{2}}^{N-1}} p_{\frac{k+m}{2}}^{N-1} \right) \\ \text {(ii)}&  \quad v_m^N = \alpha _m \left( -\mu p_m^{N} + \sum _{l=1}^{m-1} \left( \frac{\zeta }{2} - \frac{\xi \omega ^2}{2} (m-l)(2m-l) \right) v_l^{N-1} v_{m-l}^{N-1} \right. \\ &  \quad \left. + \sum _{k=m+2:2}^{2(N-1)-m} \left( \zeta {-} \frac{\xi \omega ^2 (k^2 + 3 m^2)}{4} \right) \overline{v_{\frac{k-m}{2}}^{N-1}} v_{\frac{k+m}{2}}^{N-1} \right) \\ \end{aligned} \end{array}\right. } \end{aligned}$$

where $\alpha _m = \left( - m^2 \omega ^2 + \imath m \delta \omega _0 \omega + \omega _0^2 \right) ^{-1}$ for $m \in \left\{ 1, \ldots N\right\}$. Now $v_m^N$ can be eliminated explicitly by substituting the second equation into the first, leading to a system of inhomogeneous Helmholtz equations.

## Time Discretization Via a Multiharmonic Ansatz for Complex Fields

In this section, we formally investigate multiharmonic algorithms based on neglecting the fact that the excitation and solution fields should be real-valued. We look for approximate solutions in

$$ \tilde{X}_N := \left\{ \sum _{m=0}^N \exp ( \imath m \omega t) u_m^N(x) \, : \, u_m^N \in H^2(\Omega ; \mathbb {C}) \right\} . $$

This approach, adopted also in the investigation of the (de-coupled) Westervelt equation in [21], will allow us to arrive at a simple triangular approximation scheme in the frequency domain. To this end, we simply set

26 $$\begin{aligned} g^M(x,t) = \displaystyle \sum _{m=0}^{M}\exp (\imath \omega m t) h_m^M(x), \quad \omega = \frac{2 \pi }{T}, \end{aligned}$$

and make the following Ansatz:

27 $$\begin{aligned} p^N(x,t)= \sum _{m=0}^{N} \exp (\imath m \omega t) p_m^N(x), \qquad v^N(x,t)= \sum _{m=0}^{N} \exp (\imath m \omega t) v_m^N(x), \end{aligned}$$

where the coefficients $p^N_m$, $v^N_m \in H^2(\Omega ; \mathbb {C})$ are determined by solving (13) with $Proj _{X_N}(\cdot )$ replaced by $Proj _{\tilde{X}_N}(\cdot )$.

### A Multiharmonic Cut-Off Algorithm

We next set up a simplified algorithm (compared to the one in Proposition 2) in the sense that the right-hand side in the resulting system will contain only one summation term, such that there is reduced coupling across harmonics, making it computational more efficient.

In this complex setting, for $v_0^N$, we obtain the quadratic equation

$$\begin{aligned} \omega _0^2 v_0^N - \zeta (v_0^N)^2 = 0 \end{aligned}$$

and choose the zero solution, such that we have $v_0^N=0$ as before in (25). This choice is consistent with the formulation used throughout the numerical simulations and avoids introducing spurious constant components.

#### Proposition 4

Let the assumptions of Theorem 1 hold with the acoustic source term $h=g_{tt}$, where *g* is given in (26). Under the Ansatz in (27) with $p^N_0=v^N_0=0$, the problem in (13) with $Proj _{X_N}(\cdot )$ replaced by $Proj _{\tilde{X}_N}(\cdot )$ can be rewritten as follows:

$$\begin{aligned} m= 1:&{\left\{ \begin{array}{ll} \begin{aligned} \text {(i)} &  \quad - p_1^N- \frac{c^2 + \imath \omega b }{\omega ^2} \Delta p^N_1 + c^2 \rho _0 n_0 v_1^N = -h_1^N \\ \text {(ii)} &  \quad \frac{1}{\alpha _1} v_1^N + \mu p_1^N =0\\ \end{aligned} \end{array}\right. } \\ m = \left\{ 2, \ldots , N \right\} :&{\left\{ \begin{array}{ll} \begin{aligned} \text {(i)} &  \quad - p_m^N - \frac{(c^2 + \imath m \omega b)}{m^2 \omega ^2} \Delta p_m^N + c^2 \rho _0 n_0 v_m^N \\ &  \qquad =- h_m^N- \eta \sum _{l=1}^{m-1} p_l^N p_{m-l}^N \\ \text {(ii)} &  \quad \frac{1}{\alpha _m} v_m^N + \mu p_m^N\\ &  \quad = \sum _{l=1}^{m-1} \left( \zeta - \xi \omega ^2 (m-l)(2m-l) \right) v_{l}^Nv^{N}_{m-l}, \end{aligned} \end{array}\right. } \end{aligned}$$

where $\alpha _m = \left( -m^2\omega ^2 + \imath m \delta \omega _0 \omega + \omega _0^2\right) ^{-1}$ for $m \in \left\{ 1, \ldots N\right\}$. Additionally, each of the individual functions $p_m^N$, where $m \in \left\{ 0, \ldots , N \right\}$, should satisfy boundary conditions (14).

#### Proof

Plugging the approximation given in (27) into (3) yields

$$\begin{aligned} \sum _{m=0}^{N}&\left[ - m^2 \omega ^2 p_m^N- (c^2 + \imath m \omega b) \Delta p_m^N + c^2 \rho _0 n_0 m^2 \omega ^2 v_m^N + m^2\omega ^2 h_m^N\right. \\&\left. \qquad \qquad + \sum _{l=0}^m \eta m^2 \omega ^2 p_l^N p_{m-l}^N \right] \exp (\imath m \omega t)= 0. \end{aligned}$$

Concerning the nonlinear term on the right hand-side of the ODE, we obtain

$$\begin{aligned} \text {Proj}_{\tilde{X}_N}(2 v^Nv^N_{tt} + (v^N_t)^2)&= - \sum _{m=0}^N \sum _{l=0}^m \left( 2(m-l)^2 + l (m-l) \right) \omega ^2 v_l^N v_{m-l}^N \exp (\imath m \omega t) \\&= - \sum _{m=0}^N \sum _{l=0}^m (m-l)(2m-l) \omega ^2 v_l^N v_{m-l}^N \exp (\imath m \omega t). \end{aligned}$$

Therefore, the approximation for the ODE is given by

$$\begin{aligned} \sum _{m=0}^{N}&\left[ \phantom {\sum _{l=0}^m} \left( -m^2 \omega ^2 + \delta \omega _0 \imath m \omega + \omega _0^2 \right) v_m^N + \mu p_m^N \right. \\&\left. + \sum _{l=0}^m \left( - \zeta + \xi \omega ^2 (m-l)(2m-l)\right) v_l^N v_{m-l}^N \right] \exp (\imath m \omega t) = 0. \end{aligned}$$

Using linear independence of the functions $t \mapsto \exp (\imath m \omega t)$, we immediately arrive at the iterative coupled system. $\square$

### A Linearized Multiharmonic Cut-Off Algorithm

We next also want to look at the linearized set-up given in (15) (with $Proj _{X_N}(\cdot )$ replaced by $Proj _{\tilde{X}_N}(\cdot )$) in the setting of complex solution fields. In this setting, for $u^N \in \tilde{X}_N$, we formally define $u_m^N = 0$ for all $m>N$.

#### Proposition 5

Let the assumptions of Theorem 1 hold with the acoustic source term $h=g_{tt}$, where *g* is given in (26). Under the Ansatz in (27) with $p^N_0=v^N_0=0$, the problem in (15), with $Proj _{X_N}(\cdot )$ replaced by $Proj _{\tilde{X}_N}(\cdot )$, can be rewritten as follows:

28 $$\begin{aligned} \begin{aligned} m= 1:&{\left\{ \begin{array}{ll} \begin{aligned} \text {(i)} &  \quad - p_1^N- \frac{c^2 + \imath \omega b }{\omega ^2} \Delta p^N_1 + c^2 \rho _0 n_0 v_1^{N} = -h_1^N, \\ \text {(ii)} &  \quad \frac{1}{\alpha _1} v_1^N + \mu p_1^{N} =0, \\ \end{aligned} \end{array}\right. } \\ m = \left\{ 2, \ldots , N \right\} :&{\left\{ \begin{array}{ll} \begin{aligned} \text {(i)} &  \quad - p_m^N - \frac{(c^2 + \imath m \omega b)}{m^2 \omega ^2} \Delta p_m^N + c^2 \rho _0 n_0 v_m^{N} \\ &  \quad =- h_m^N-\eta \sum _{l=1}^{m-1} p_l^{N-1} p_{m-l}^{N-1}, \\ \text {(ii)} &  \quad \frac{1}{\alpha _m} v_m^N + \mu p_m^{N} \\ &  \quad = \sum _{l=1}^{m-1} \left( \zeta - \xi \omega ^2 (m-l)(2m-l) \right) v_{l}^{N-1}v^{N-1}_{m-l}, \end{aligned} \end{array}\right. } \end{aligned} \end{aligned}$$

where $\alpha _m = \left( -m^2\omega ^2 + \imath m \delta \omega _0 \omega + \omega _0^2\right) ^{-1}$ for $m \in \left\{ 1, \ldots N\right\}$. Additionally, each of the individual functions $p_m^N$ for $m \in \left\{ 0, \ldots , N \right\}$ is supposed to satisfy boundary conditions (14).

#### Proof

Similarly to the previous case, substituting the approximation provided in (27) into (3) immediately yields the iterative coupled system. $\square$

We see that when working with complex fields from the beginning, we end up with a lower triangular system of inhomogeneous Helmholtz equations and algebraic equations that can be solved by substitution.

#### Remark 1

*(On the assumption of complex fields)* In this section, the approximations $(p^N,v^N)$ are complex-valued, so convergence could only be studied in the complex counterpart of $X_N\times X_N$. This does not by itself imply that $\Re (p^N,v^N)\rightarrow (p,v)$ as $N\rightarrow \infty$; such a conclusion would require enforcing the reality constraint (symmetric $\pm m$ spectrum with $\hat{u}_{-m}=\overline{\hat{u}_m}$). Following [3], one may formulate the multiharmonic Ansatz with complex Fourier coefficients (omitting the explicit $\Re$ in the time reconstruction), but the resulting nonlinear maps are not complex-differentiable (holomorphic). Hence a Newton scheme in the sense of complex analysis is not applicable; in practice one uses the real formulation (or a split into real/imaginary parts) despite the algebraic brevity of the complex notation.

For clarity, eq. (25) (real basis) contains both sum- and difference-frequency couplings generated by the quadratic terms, visible in the two convolution sums $\sum _{l=1}^{m-1} p_l\,p_{m-l}$ and $\sum _{k=m+2:2}^{2(N-1)-m} p_{(k-m)/2}\,p_{(k+m)/2}$. In contrast, the complex projection (28) is posed on the half-spectrum $\{e^{\mathrm i m\omega _0 t}\}_{m=1}^N$ without imposing $\hat{u}_{-m}=\overline{\hat{u}_m}$; only the sum-frequency interactions remain and the block system becomes lower-triangular (solvable by substitution). If one augments the complex space to include $\pm m$ and enforces the reality constraint, the complex and real formulations are algebraically equivalent. In our parameter regime, the neglected difference-frequency contributions are numerically negligible, which explains the agreement observed in Fig. 3 in Section 6.

### A Simple Two-Harmonic Approximation Scheme

In certain imaging applications, the fundamental frequency and the second harmonic are considered the most significant; see, for example, [31]. We thus compare the multiharmonic iterative scheme with a simplified two-harmonic scheme to evaluate the extent of information loss.

For two harmonics, that is, for $N=2$ with $p_0^{(2)}=0$ and $v_0^{(2)}=0$, the approximations for the pressure *p* and the volume *v* are given by

$$\begin{aligned} \begin{aligned} p(x,t) \approx p^{(2)}(x,t)&= p_1^{(2)}(x) \exp (\imath \omega t) + p_2^{(2)}(x) \exp (2 \imath \omega t), \\ v(x,t) \approx v^{(2)}(x,t)&= v_1^{(2)}(x) \exp (\imath \omega t) + v_2^{(2)}(x) \exp (2 \imath \omega t). \end{aligned} \end{aligned}$$

The corresponding scheme for computing $p_{1,2}^{(2)}$ and $v_{1,2}^{(2)}$ can be found by setting $N=2$ in Proposition 4. This results in the system

29 $$\begin{aligned} \begin{aligned} m= 1:&{\left\{ \begin{array}{ll} \begin{aligned} \text {(i)} &  \quad p_1^{(2)}+ \frac{c^2 + \imath \omega b }{\omega ^2} \Delta p^{(2)}_1 - c^2 \rho _0 n_0 v_1^{(2)} = h_1^{(2)}, \\ \text {(ii)} &  \quad \frac{1}{\alpha _1} v_1^{(2)} + \mu p_1^{(2)} =0,\\ \end{aligned} \end{array}\right. } \\ m = 2:&{\left\{ \begin{array}{ll} \begin{aligned} \text {(i)} &  \quad p_2^{(2)}+ \frac{c^2 + 2 \imath \omega b}{4 \omega ^2} \Delta p_2^{(2)} - c^2 \rho _0 n_0 v_2^{(2)} = h_2^{(2)} +\frac{ \beta _a }{ \rho _0 c^2} (p_1^{(2)})^2, \\ \text {(ii)} &  \quad \frac{1}{\alpha _2} v_2^{(2)} + \mu p_2^{(2)} = \left( \zeta - 3 \xi \omega ^2 \right) (v_1^{(2)})^2, \end{aligned} \end{array}\right. } \end{aligned} \end{aligned}$$

where, as before,

$$\begin{aligned} \alpha _m = \frac{1}{- m^2 \omega ^2 + \imath m \delta \omega _0 \omega + \omega _0^2} = \frac{ \omega _0^2- m^2 \omega ^2}{ (\omega _0^2- m^2 \omega ^2 )^2+ (m \delta \omega _0 \omega )^2} -\imath \frac{ m \delta \omega _0 \omega }{ (\omega _0^2- m^2 \omega ^2 )^2+ (m \delta \omega _0 \omega )^2} \end{aligned}$$

for $m \in \{1,2\}$. Above, we have rewritten $\eta$ as $\eta = \dfrac{ \beta _a }{ \rho _0 c^2}$ to make the influence of the nonlinearity parameter in the medium $\beta _a$ on the system explicit. In this two-harmonic setting, we can express $v_1^{(2)}$ in terms of $p_1^{(2)}$ and $v_2^{(2)}$ in terms of $p_2^{(2)}$:

$$\begin{aligned} \begin{aligned} v_1^{(2)}&= - \alpha _1 \mu \, p_1^{(2)}, \\ v_2^{(2)}&= \alpha _2 \left[ - \mu p_2^{(2)} + (\zeta -3\xi \omega ^2) {\alpha }_1^2 \mu ^2 \left( p_1^{(2)}\right) ^2 \right] . \end{aligned} \end{aligned}$$

We can then use these expressions to eliminate $v_1^{(2)}$ and $v_2^{(2)}$ for the first and third equations in (29). Multiplying the resulting equations by $m^2 \omega ^2/c^2$ yields the following system for the two pressure coefficients:

30 $$\begin{aligned} \begin{aligned} m = 1: \qquad&\frac{ \omega ^2}{\tilde{c}_1^2} p_1^{(2)} +\left( 1 + \imath \frac{ b \omega }{c^2} \right) \Delta p_1^{(2)} = \frac{\omega ^2}{c^2} h_1^{(2)},\\ m = 2: \qquad&\frac{4 \omega ^2}{\tilde{c}_2^2} p_2^{(2)} + \left( 1 +\imath \frac{2 b \omega }{c^2} \right) \Delta p_2^{(2)} = \frac{4 \omega ^2}{c^2} h_2^{(2)} + \frac{4 \omega ^2 }{\rho _0 c^4 } \left( \beta _a + \tilde{\beta }_a\right) \left( p_1^{(2)}\right) ^2, \end{aligned} \end{aligned}$$

where

31 $$\begin{aligned} \frac{1}{\tilde{c}_{m}^2} = \frac{1}{c^2} + \rho _0 n_0 \mu \alpha _{m}, \qquad \tilde{\beta }_a= c^4 \rho _0^2 n_0(\zeta -3\xi \omega ^2)\mu ^2 \alpha _1^2 \alpha _2. \end{aligned}$$

The above system clearly displays the influence of microbubbles on wave propagation, particularly through their impact on the effective wave speed and the nonlinearity parameter.

- From equations (30), we see that the effective wave number in the presence of microbubbles in a two-harmonic setting is
  $$\begin{aligned} \tilde{k}_m= \frac{m \omega }{\tilde{c}_m}, \quad \text {with } \tilde{c}_m \ \text { given in } (31). \end{aligned}$$
  Note that $\tilde{k}_m$ is in general complex since $\alpha _m$ is complex, and thus the attenuation in the wave propagation is due both to the acoustic dissipation via the $\imath \frac{m b \omega }{c^2} \Delta p_m^{(2)}$ terms as well as the presence of microbubbles via the $\imath \Im (\tilde{k}^2_m) p_m^{(2)}$ terms.
- In the second equation in (30), we see the influence of microbubbles on the nonlinearity parameter as their presence leads to the additional $\tilde{\beta }_a$ term on the right-hand side, thus effectively enlarging the nonlinearity parameter $\beta _a$. By setting $\tilde{\beta } = 0$ and replacing $\tilde{c}^2_{m}$ with $c^2$, the system reduces to the two-harmonic expansion of Westervelt’s equation for a strongly damped medium without bubbles.

## Numerical Experiments

In this section, we investigate numerically the algorithms set up in Sections 3 and 5. To discretize the resulting Helmholtz problems, we use a conforming finite element method. We note that algorithmic frameworks of the multiharmonic-FEM type used in this section are known as the Harmonic Balance Finite Element Method (HBFEM) in the context of electromagnetism; see, e.g., [3, 4, 27].

The discretization of the classical Helmholtz equation $-\Delta u-k^2 u=f$ with $k>0$, and the issues related to the so-called pollution effect and sign-indefiniteness have been extensively studied in the literature; see, e.g., [2, 19, 29, 30], and the references contained therein. Recall that the Helmholtz problems appearing in linearized problems in (25) and (28) have the general form

32 $$\begin{aligned} \left\{ \begin{aligned}&-\left( 1+\imath \omega \frac{m b}{c^2} \right) \Delta u - (k^2+\mathfrak {a}) u + \imath \mathfrak {b} u= f_\omega \quad \text { in }\, \Omega ,\quad \text {with } k= \frac{m \omega }{c},\\&(\imath \omega m \beta + \gamma ) u + \nabla u \cdot \boldsymbol{n}= 0 \quad \text { on }\, \partial \Omega , \end{aligned} \right. \end{aligned}$$

for a known right-hand side $f_\omega \in L^2(\Omega ; \mathbb {C})$ (that depends on $\omega$), and with

$$\begin{aligned} \mathfrak {a}= \mu \rho _0 n_0 \frac{m^2 \omega ^2 (\omega _0^2 - m^2 \omega ^2)}{(\omega _0^2-m^2\omega ^2)^2 + (m \delta \omega _0 \omega )^2}, \quad \mathfrak {b}= \mu \rho _0 n_0 \frac{m^3 \omega ^3 \delta \omega _0 }{(\omega _0^2-m^2\omega ^2)^2 + (m \delta \omega _0 \omega )^2}. \end{aligned}$$

The variational form of (32) is given by

33 $$\begin{aligned} a(u, \phi ) = l(\phi ) \quad \text {for all } \phi \in H^1(\Omega ; \mathbb {C}), \end{aligned}$$

where the sesquilinear form $a: H^1(\Omega )\times H^1(\Omega )\rightarrow \mathbb {C}$ is defined asand $l(\phi ) :=\int _{\Omega }f \overline{\phi }\, d x$. We then have

$$\begin{aligned} \begin{aligned} \mathfrak {R}\left( a(u,u)\right) = \Vert \nabla u\Vert ^2_{L^2(\Omega )} - (k^2+\mathfrak {a})\Vert u\Vert ^2_{L^2(\Omega )} + \left( \gamma - \frac{b\beta (m \omega )^2}{c^2}\right) \Vert u\Vert ^2_{L^2(\partial \Omega )}. \end{aligned} \end{aligned}$$

and

$$\begin{aligned} \begin{aligned} \Im \left( a(u,u)\right) = \frac{m \omega b}{c^2} \Vert \nabla u\Vert ^2_{L^2(\Omega )} +\mathfrak {b}\Vert u\Vert ^2_{L^2(\Omega )}+\left( \gamma \frac{b}{c^2}+\beta \right) \omega m \Vert u\Vert ^2_{L^2(\partial \Omega )} \end{aligned} \end{aligned}$$

Therefore, we can conclude thatwhich showcases how the coercivity of $a(\cdot , \cdot )$ is influenced by the relative behavior of the various medium and frequency-dependent parameters. Note that from the complex part of (33), we obtain the bound

$$\begin{aligned} \frac{m \omega b}{c^2} \Vert \nabla u\Vert ^2_{L^2(\Omega )} +\frac{\mathfrak {b}}{2} \Vert u\Vert ^2_{L^2(\Omega )}+\left( \gamma \frac{b}{c^2}+\beta \right) \omega m \Vert u\Vert ^2_{L^2(\partial \Omega )} \le \frac{1}{2 \mathfrak {b}}\Vert f_\omega \Vert ^2_{L^2(\Omega )}, \end{aligned}$$

which carries over to the finite element setting.

### Numerical Framework

All simulations are performed using a conforming two-dimensional finite element discretization with linear Lagrange elements, using FEniCSx v0.9.0 (see, e.g., [5]) with Gmsh [13] employed for meshing. A direct LU factorization is used to solve the resulting linear systems without iterative refinement.^1^

We use a circular domain of propagation $\Omega = B_{0.2}(0) \subset \mathbb {R}^2$ with a radius of $0.2 \, {\text {m}}$ and place a monopole source $h=h(x)$ at $x_0 = (0,0)^T$ defined as

$$\begin{aligned} h(x) = {\left\{ \begin{array}{ll} \frac{a}{4 r_\delta } \left( 1 + \cos \left( \pi \frac{\left\| x - x_0 \right\| _{l^2} }{2 r_{\delta }}\right) \right) \qquad \quad &  \left\| x - x_0 \right\| _{l^2} \le 2 r_{\delta }, \\ 0 &  \text {otherwise.} \end{array}\right. } \end{aligned}$$

We present numerical results for various values of *a*, using $\omega =\omega _0$ and $r_{\delta }=0.004$ unless stated otherwise. The physical parameters used in the simulations are chosen as typical in ultrasound contrast imaging (see, e.g., [17, 18]), and are listed in Table 1.

**Table 1:** Overview of the parameter values used in the simulations

*c*	speed of sound	1500 m/s	*b*	diffusivity of sound	$1 \cdot 10^{-3}$
$\rho _0$	mixture mass density	$1000\, kg/\hbox {m}^{3}$	$\beta _a$	nonlinearity coefficient	3.5
$R_0$	initial radius	$2 \,\upmu \hbox {m}$	$n_0$	bubble number density	$1 \cdot 10^{12} \, 1/\hbox {mL}$
$P_0$	vapor pressure	100 Pa	$\kappa$	adiabatic exponent	1.4
$\nu$	kinematic viscosity	$8.9 \cdot 10^{-6} \hbox {m}^{2}/\hbox {s}$			

Using the values in Table 1, we compute

$$\begin{aligned} \omega _0 = \sqrt{\frac{3 \kappa P_0}{\rho _0 R_0^2}} \approx 0.324 \, {\text {M}\text {Hz}} \end{aligned}$$

and additionally $\delta = \frac{4 \nu }{\omega _0 R_0^2}$, $v_0 = \frac{4 \pi }{3} R_0^3$, $\mu = \frac{4 \pi R_0}{\rho _0}$, $\zeta = \frac{(\kappa + 1) \omega _0^2}{2 v_0}$, and $\xi = \frac{1}{6 v_0}$ are fixed. Furthermore, we set $\beta = \frac{1}{c}$ and $\gamma = 1$ in the boundary conditions for Helmholtz problems.

### The Influence of Spatial Discretization

For a fixed *N*, we first investigate the convergence of a quantity of interest given by

$$\begin{aligned} \left\| \Re (p_{h_{FEM }}^{N}) \right\| _{L^{\infty }(0,T; L^2(\Omega ))}, \end{aligned}$$

where $p_{h_{FEM }}^N$ denotes the approximate pressure, as the spatial discretization parameter $h_{FEM }\searrow 0$. A common rule of thumb for finite element discretizations of the classical Helmholtz equation with linear elements is to use at least 10 elements per wavelength in each spatial direction. If the mesh is not refined accordingly, one encounters the above-mentioned pollution effect, where numerical phase errors grow with increasing *k*; see, e.g., [2, 19, 29].

**Fig. 2:**
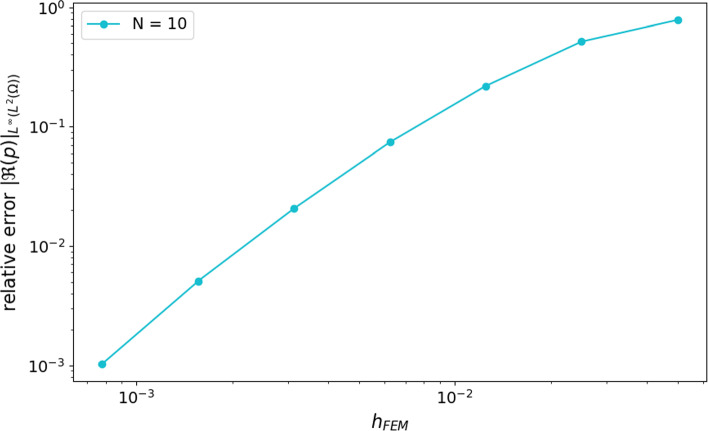
Relative error (34) of the quantity of interest with respect to the mesh size $h_{FEM }$ on a log–log scale

Figure 2 illustrates the behavior of the relative difference

34 $$\begin{aligned} \frac{ \left| \bigl \Vert \Re (p_{h_{FEM }}^{N})\bigr \Vert _{L^\infty (0,T;L^2(\Omega ))} - \bigl \Vert \Re (p^{\textrm{ref}})\bigr \Vert _{L^\infty (0,T;L^2(\Omega ))} \right| }{ \bigl \Vert \Re (p^{\textrm{ref}})\bigr \Vert _{L^\infty (0,T;L^2(\Omega ))} }, \end{aligned}$$

as the mesh is refined, using the solution on the finest spatial mesh as reference. A clear algebraic decay is observed for decreasing mesh size $h_{FEM }$, indicating that the spatial discretization error is well controlled in the considered parameter regime and that the chosen mesh resolutions are sufficient to avoid significant pollution effects for the frequencies under consideration. The similarity of the convergence curves for different truncation levels *N* shows that the spatial discretization error dominates over the effect of the multiharmonic truncation.

**Table 2:** Mesh sizes, degrees of freedom, and computational time for different spatial discretizations

$h_{FEM }$	Nodes	Elements	Time
0.000390625	9,564,795	1,933,589	9984.8 s
0.00078125	239,024	478,047	1244.3 s
0.0015625	60,136	120,271	145.5 s
0.003125	15,218	30,435	19.8 s
0.00625	3,896	7,791	5.16 s
0.0125	1,010	2,019	2.72 s
0.025	279	557	2.24 s
0.05	85	169	2.15 s

Additional quantitative data are provided in Table 2, which lists the corresponding mesh sizes, number of nodes and elements, as well as the computational time required for each case. This highlights the significant increase in computational cost associated with finer meshes, especially for very small $h_{FEM }$, where both the number of degrees of freedom and the runtime grow substantially. All simulations were carried out on a standard laptop (Intel Core i7, 16GB RAM, no dedicated GPU), underscoring the feasibility of the method even without access to high-performance computing resources.

In the following simulations, we use a mesh size of $h_{FEM }= 0.003$. Unless stated otherwise in the figure captions, we set $r_{\delta } = 0.004$, $a = 10^5$, and $\omega = \omega _0$.

### Comparison of Real- and Complex-Valued Fields

We compare the five-harmonic pressure obtained with the linearized multiharmonic cut-off algorithm using the complex-valued formulation (28) and the real-valued formulation (25). In the considered numerical setting, no noticeable differences between the resulting pressure fields are observed.

**Fig. 3:**
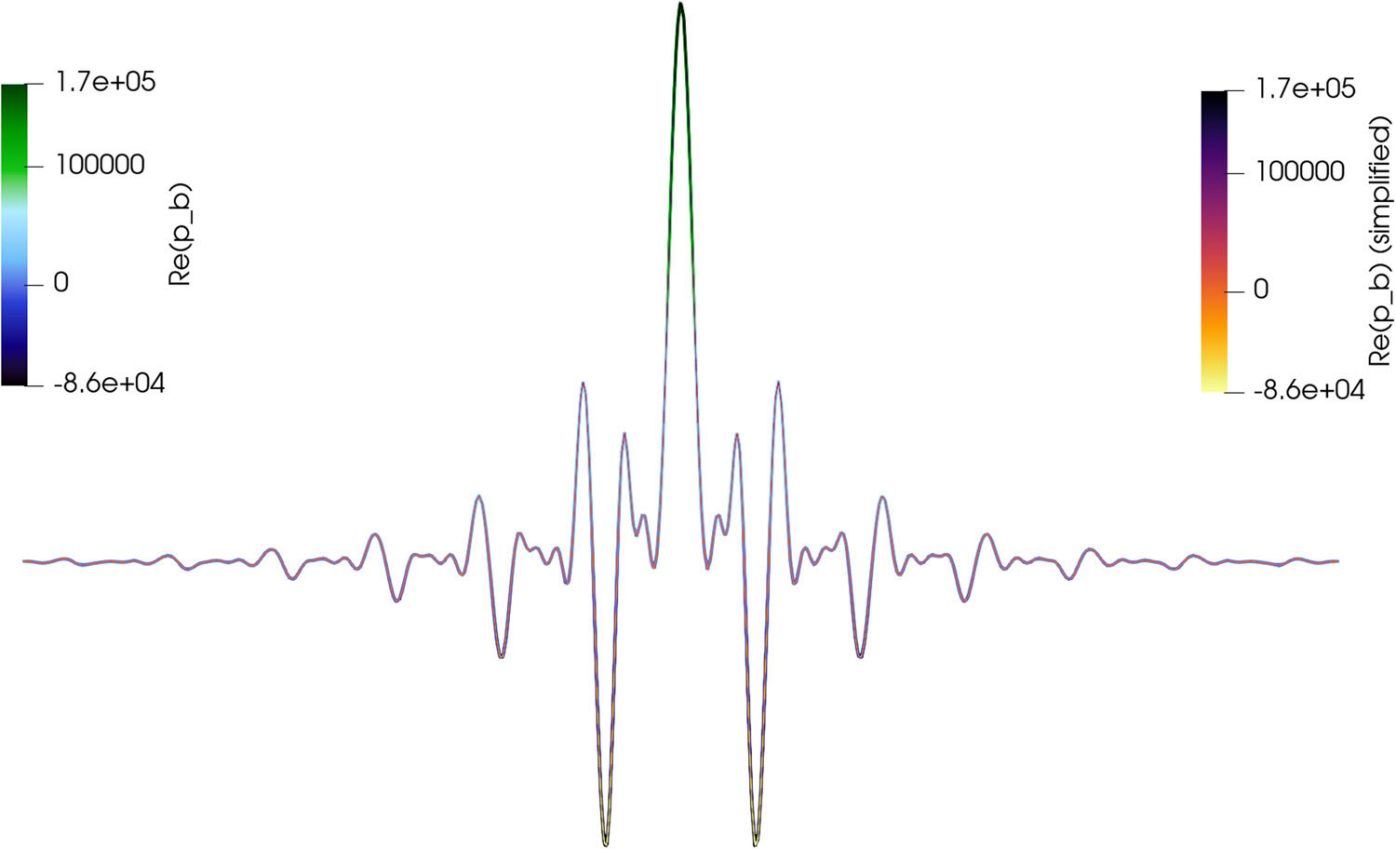
Five-harmonic expansion $\mathfrak {R}(p_{h_{FEM }}^{(5)}(x_1,x_{2,0},t_0))$ plotted as a function of $x_1$ for a fixed $x_2=x_{2,0}$ and $t=t_0$ resulting from complex fields in (28) vs. real setting in (25)

To further quantify this observation, we compare the $L^2(\Omega )$-norms of the harmonic components obtained from the complex- and real-valued formulations. The relative differences in these norms are below $5 \cdot 10^{-3}$ for the dominant harmonics $m = 1,\ldots ,4$, and below $2 \cdot 10^{-2}$ for higher harmonics $m \ge 5$. At the same time, the amplitudes of these higher harmonics are already several orders of magnitude smaller than that of the fundamental mode.

We therefore consider both formulations to be numerically equivalent in the considered parameter regime. Since the complex formulation leads to a computationally more efficient algorithm, it is used in all subsequent simulations.

### The Influence of the Number of Harmonics

We next analyze how many harmonics need to be retained in the multiharmonic expansion for the considered parameter settings. We first investigate the behavior of the approximate pressure field with respect to the truncation level *N* for a fixed $h_{FEM }$, which motivates the choice of a suitable reference solution.

**Fig. 4:**
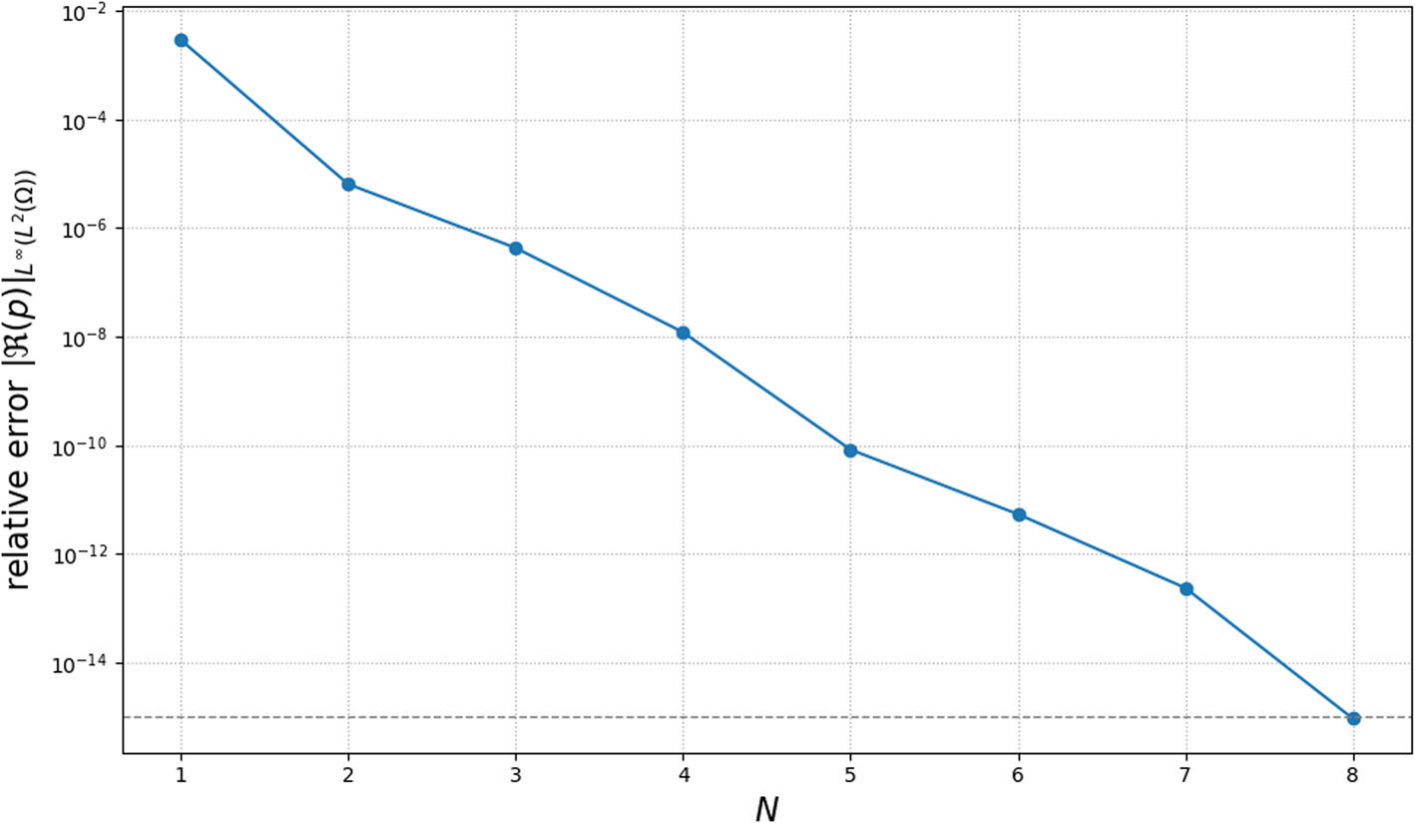
Relative error (34) of the quantity of interest with respect to the truncation level *N* on a semi-log scale (logarithmic *y*-axis)

Figure 4 shows the relative error of the pressure field as defined in (34) for increasing truncation levels *N*, using the solution with $N=10$ harmonics as reference. For larger values of *N*, the relative error levels off at approximately machine precision, reflecting that further changes in the norm are below the accuracy of the numerical discretization and floating-point arithmetic.

To illustrate how the contributions from individual harmonics are reflected in the approximate pressure field, we additionally consider the effect of truncating the multiharmonic expansion in the time domain. For a fixed reference time $t_0$, we compute the pointwise differences

$$ \left| p_{h_{FEM }}^N(x,t_0) -p_{h_{FEM }}^{(10)}(x,t_0) \right| , $$

where $p_{h_{FEM }}^N(x,t)$ denotes the approximate pressure field obtained using the first *N* harmonics and the solution with $N=10$ harmonics serves as reference.

**Fig. 5:**
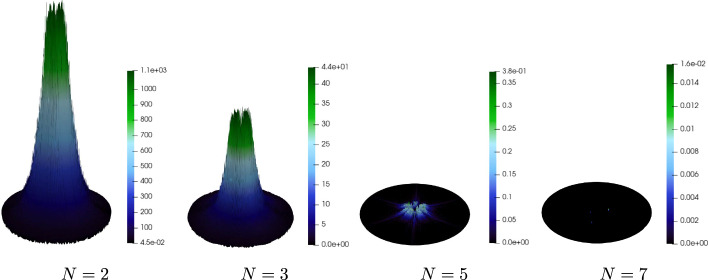
Pointwise differences $|p_{h_{FEM }}^N(x,t_0) - p_{h_{FEM }}^{(10)}(x,t_0) |$ for $a=10^5$ at a fixed time $t_0$ and different truncation levels $N=2,3,5,7$

Figure 5 visualizes the spatial structure of the truncation error for different truncation levels *N* in the strongly nonlinear case $a=10^5$. For small values of *N*, the deviations from the reference solution are large and extend over significant parts of the domain, whereas increasing the number of retained harmonics leads to a rapid reduction and spatial localization of the differences. In particular, once approximately five harmonics are included, the remaining discrepancies become small throughout the domain, which is fully consistent with the harmonic-wise decay observed in Table 3.

We now fix the truncation index to $N=10$ and examine the harmonic content of the numerical solution in more detail. To this end, we compute the $L^2(\Omega )$-norms $\Vert p^N_{h_{FEM }, m}\Vert _{L^2(\Omega )}$ of the harmonic coefficients and normalize them by the fundamental mode,

$$ r_m(a) := \frac{\Vert p^N_{h_{FEM }, m}\Vert _{L^2(\Omega )}}{\Vert p^N_{h_{FEM },1}\Vert _{L^2(\Omega )}}, $$

for each driving amplitude *a* and harmonic index *m*.

From Table 3 we observe that the relative amplitudes of higher harmonics decrease rapidly with increasing *m*, and that this decay becomes more pronounced as the driving amplitude *a* is reduced, indicating a weaker nonlinear response. For $a=10^3$, the dominant contribution comes from the first two harmonics, while for $a=10^4$ the first three harmonics contribute noticeably. In the strongly nonlinear case $a=10^5$, appreciable contributions extend to higher harmonic indices, indicating that retaining at most four to five harmonics is appropriate to accurately represent the pressure field.

**Table 3:** Relative $L^2(\Omega )$-norms of higher harmonics $r_m(a)$ for $m=2,\dots ,5$ and $N=10$

*a*	$r_2(a)$	$r_3(a)$	$r_4(a)$	$r_5(a)$
$10^3$	$2.23\cdot 10^{-4}$	$8.11\cdot 10^{-8}$	$2.94\cdot 10^{-11}$	$1.15\cdot 10^{-14}$
$10^4$	$2.23\cdot 10^{-3}$	$8.11\cdot 10^{-6}$	$2.94\cdot 10^{-8}$	$1.15\cdot 10^{-10}$
$10^5$	$2.23\cdot 10^{-2}$	$8.11\cdot 10^{-4}$	$2.94\cdot 10^{-5}$	$1.15\cdot 10^{-6}$

### Comparison of the Pressure Field with and Without Bubbles

Finally, we compare the pressure field obtained from the multiharmonic formulation in the presence and absence of bubble dynamics in order to assess how bubble coupling affects the nonlinear response of the system. We examine the pressure field in the time domain and the harmonic coefficients.

**Fig. 6:**
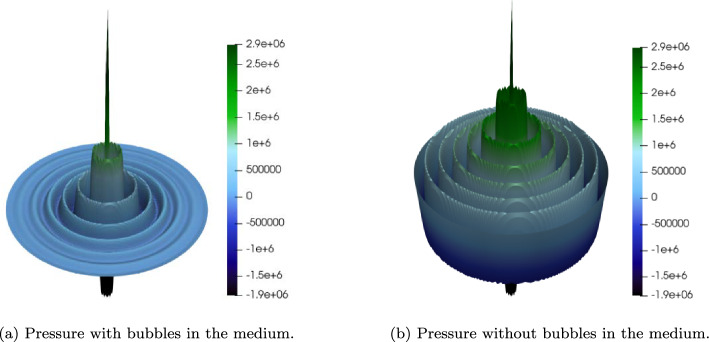
Pressure field $\Re (p_{h_{FEM }}^{(5)}(x,t_0))$ at a fixed reference time $t_0$ for $a=10^5$, with and without bubbles in the medium

Figure 6 compares the real part of the pressure distribution within the domain at a specific time point with and without the presence of bubbles in the medium; that is, the real part of the pressure obtained using the algorithm in (30) and the same algorithm with $n_0=\tilde{\beta }=0$. The presence of bubbles leads to an overall damping effect, resulting in a reduced pressure amplitude across the domain. This observation aligns with the behavior predicted by the system of equations in (30), as the modified speed of sound introduces attenuation. Although the source amplitude is fixed in this case, we note that the differences become more pronounced for larger source amplitudes, leading to higher overall pressure levels.

To quantify these differences, we compare the harmonic content of the pressure field in both settings. For a fixed truncation level $N=10$, we consider the $L^2(\Omega )$-norms of the pressure harmonics $\Vert p_{h_{FEM },m}^N\Vert _{L^2(\Omega )}$ and report their relative contributions with respect to the fundamental mode,

$$ r_m := \frac{\Vert p_{h_{FEM },m}^N\Vert _{L^2(\Omega )}}{\Vert p_{h_{FEM },1}^N\Vert _{L^2(\Omega )}}. $$

Table 4 shows that higher pressure harmonics have systematically larger relative amplitudes in the absence of bubbles. In particular, the decay of the relative harmonic ratios is significantly slower in the bubble-free case, with comparable contributions persisting over a broader range of harmonic indices. When bubble dynamics are included, the relative amplitudes of higher pressure harmonics decrease much more rapidly, dropping below $10^{-6}$ already around the fifth harmonic. In addition, the harmonic coefficients associated with the bubble variable $v_m$ are several orders of magnitude smaller than the corresponding pressure harmonics for all *m*, indicating that nonlinear effects transferred into the bubble dynamics are strongly damped.

**Table 4:** Relative $L^2(\Omega )$-norms $r_m$ of the pressure harmonics for $a = 10^5$ with and without bubbles in the medium ($N=10$)

	$r_2$	$r_3$	$r_4$	$r_5$	$r_6$
with bubbles	$2.23\cdot 10^{-2}$	$8.11\cdot 10^{-4}$	$2.94\cdot 10^{-5}$	$1.15\cdot 10^{-6}$	$1.13\cdot 10^{-7}$
without bubbles	$5.48\cdot 10^{-2}$	$1.78\cdot 10^{-3}$	$5.50\cdot 10^{-5}$	$2.61\cdot 10^{-6}$	$2.64\cdot 10^{-6}$

We further examine the temporal evolution of the five-harmonic expansion at different spatial points in this context.

**Fig. 7:**
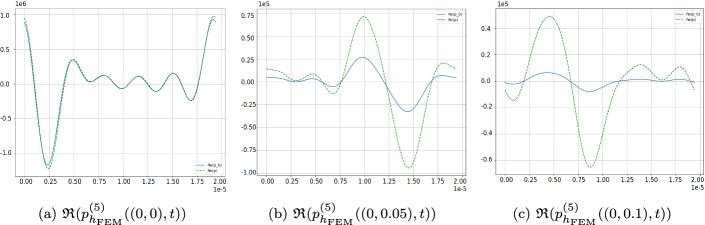
Real part of the pressure from the five-harmonic expansion computed using (28) without bubbles ($n_0=0$, dashed green line) and with bubbles (blue line) over time at three spatial points

**Fig. 8:**
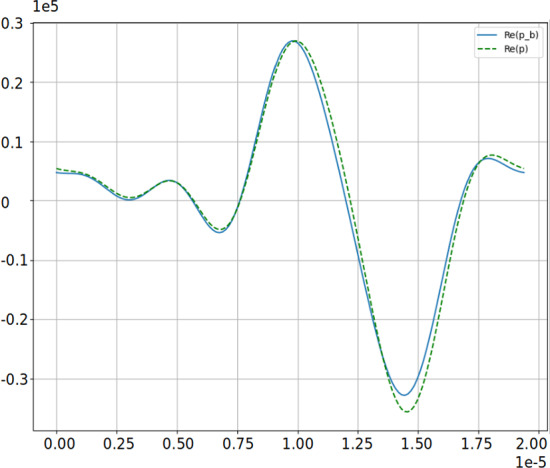
Real part of the pressure $\mathfrak {R}({p_{h_{FEM }}^{(5)}(0,0.05,t)})$ from the five-harmonic expansion computed using (28) without bubbles ($n_0=0$, dashed green line) and with bubbles (blue line, scaled to the maximum of the green curve) over time

As illustrated in Figure 7, the presence of microbubbles near the source has little effect on the pressure waves. However, as the distance from the source increases, the signal strength decreases. The microbubbles not only attenuate the waveform but also enhance its nonlinear characteristics. To better visualize these effects, we scale the pressure waveform obtained from the five-harmonic expansion with bubbles to the maximum of the waveform without microbubbles, as shown in Figure 8.

### Conclusion

The numerical results demonstrate that microbubbles introduce significant attenuation and phase shifts to the wave propagation, particularly at greater distances from the source. The impact increases with microbubble density and source amplitude, and is further influenced by the driving frequency. Regarding the number of harmonics *N*, in the considered numerical settings using three to five harmonics within the simplified multiharmonic framework obtained from complex fields provides a good compromise between computational efficiency and accuracy. This relatively low number of harmonics makes the approach promising for use in practical applications.

## Outlook

In practice, nonlinear acoustic interactions between microbubbles and ultrasound waves generate not only harmonics, which are frequency components at integer multiples of the driving frequency, but also subharmonics, which appear at fractional multiples of the driving frequency, such as $\frac{\omega }{2}$, $\frac{\omega }{3}, \, \ldots$; see, e.g., [26]. Subharmonics primarily appear due to non-spherical deformations and multibubble interactions. While these effects are not included in our current model, extending the framework to incorporate them would be an interesting direction for future research.

Expanding the present theoretical and numerical framework to explore other phenomena relevant to applications of focused ultrasound waves is also of practical interest. For instance, localized heating, cavitation, and nonlocal attenuation play an important role in therapeutic ultrasound applications such as targeted drug delivery, and it would be worthwhile to investigate the potential role of multiharmonic expansions in these modeling contexts.

Furthermore, investigating inverse problems related to reconstructing spatially varying parameters, such as the bubble number density $n_0=n_0(x)$ or the nonlinearity parameter $\beta _a=\beta _a(x)$ from measured acoustic signals is an important task in the context of contrast-enhanced ultrasound imaging, as it could help improve diagnostic accuracy in the long run.

## Data Availability

No datasets were generated or analyzed during the current study.

## A Proof of Lemma 1

We present here the postponed proof of Lemma 1.

### Proof

We can write the linearized second-order ODE in the matrix system form with $V=[v \ v_t]^T$:

35 $$\begin{aligned} \begin{aligned}&V_t =\, AV + F, \quad A= \begin{bmatrix} 0 &  1 \\ -\omega _0^2 &  -\delta \omega _0 \end{bmatrix},\ F=\begin{bmatrix} 0 \\ f \end{bmatrix} \end{aligned} \end{aligned}$$

a.e. in space. Let $v^{(1)}$ and $v^{(2)}$ be to linearly independent solutions of the homogeneous equation $v_{tt}+ \delta \omega _0 v_t+ \omega _0^2 v =0$.

$$\begin{aligned} \begin{aligned} (v^{(1)}, v^{(2)}) = {\left\{ \begin{array}{ll} (e^{r_1 t}, e^{r_2 t}), \ r_{1,2} = \frac{- \delta \omega _0\pm \sqrt{\delta ^2 \omega _0^2 - 4 \omega _0^2}}{2} \quad & \text {if} \quad \delta \omega _0 > 2 \omega _0, \\ (e^{-\omega _0 t}, t e^{-\omega _0 t}), \ \quad & \text {if} \quad \delta \omega _0 = 2 \omega _0, \\ e^{- \delta \omega _0 t/2}(\cos \alpha t,\, \sin \alpha t), \ \alpha = \frac{ \sqrt{ 4 \omega _0^2-\delta ^2 \omega _0^2}}{2} \quad & \text {if} \quad \delta \omega _0 < 2 \omega _0. \end{array}\right. } \end{aligned} \end{aligned}$$

(i) Let first $\delta \omega _0 > 2 \omega _0$. Then the fundamental matrix is given by

$$\begin{aligned} \begin{aligned} Z(t) =\, \begin{bmatrix} v^{(1)}&  v^{(2)}\\ (v^{(1)})' \quad &  (v^{(2)})' \end{bmatrix}=\, \begin{bmatrix} e^{r_1 t} &  e^{r_2 t} \\ r_1 e^{r_1 t} &  r_2 e^{r_2 t} \end{bmatrix}, \end{aligned} \end{aligned}$$

and we have

$$\begin{aligned} \begin{aligned} \left( Z(t) \right) ^{-1} = \frac{1}{r_2-r_1}e^{-(r_1+r_2)t} \begin{bmatrix} r_2 e^{r_2 t} &  -e^{r_2 t} \\ -r_1 e^{r_1 t} \   &  e^{r_1 t} \end{bmatrix}, \end{aligned} \end{aligned}$$

where $r_1-r_2 = \sqrt{\delta ^2 \omega _0^2-4 \omega _0^2}$ and $r_1+r_2 = -\delta \omega _0$. Then the principal fundamental matrix is

$$\begin{aligned} \begin{aligned} X(t) = Z(t) \left( Z(0)\right) ^{-1} =&\, \frac{1}{r_2-r_1} \begin{bmatrix} e^{r_1 t} &  e^{r_2 t} \\ r_1 e^{r_1 t} &  r_2 e^{r_2 t} \end{bmatrix} \begin{bmatrix} r_2 &  -1 \\ -r_1 \   &  1 \end{bmatrix} \\ =&\, \frac{1}{r_2-r_1} \begin{bmatrix} r_2 e^{r_1t}-r_1 e^{r_2 t} &  -e^{r_1t}+e^{r_2t}\\ r_1 r_2 (e^{r_1 t} -e^{r_2t}) &  -r_1 e^{r_1t} +r_2 e^{r_2 t} \end{bmatrix}. \end{aligned} \end{aligned}$$

Note that $X_t =A X$. Furthermore,

$$\begin{aligned} \begin{aligned} (X(t))^{-1} = \frac{1}{r_2-r_1} \begin{bmatrix} r_2 e^{-r_1 t}-r_1 e^{-r_2t} &  -e^{-r_1t}+e^{-r_2t} \\ r_1 r_2 (e^{-r_1 t} - e^{-r_2t}) &  -r_1 e^{-r_1t}+r_2 e^{-r_2 t} \end{bmatrix}. \end{aligned} \end{aligned}$$

Then using the method of variation of parameters, we find that the solution of (35) has the form

36 $$\begin{aligned} \begin{aligned} V(t) = X(t)\left( V(0)+ \int _0^t\left( X(\tau )\right) ^{-1}f(\tau )\, d \tau \right) . \end{aligned} \end{aligned}$$

Since periodic solutions must satisfy $V(0)=V(T)$, we then conclude that

$$\begin{aligned} \begin{aligned} (I-X(T)) V(0)= X(T) \int _0^T \left( X(\tau )\right) ^{-1}f(\tau )\, d \tau . \end{aligned} \end{aligned}$$

We have

$$\begin{aligned} \det (I- X(T)) = 1 - (e^{r_1 T}+ e^{r_2 T}) + e^{(r_1 + r_2)T} = (1-e^{r_1 T })(1-e^{r_2 T })\ne 0. \end{aligned}$$

Therefore, since the matrix $I-X(T)$ is invertible, we find that

37 $$\begin{aligned} \begin{aligned} V(0)= (I-X(T))^{-1}X(T) \int _0^T \left( X(\tau )\right) ^{-1}f(\tau )\, d \tau . \end{aligned} \end{aligned}$$

Furthermore, from (36) and (37), we have the following bound:

$$\begin{aligned} \begin{aligned} \Vert V\Vert _{L^\infty (L^\infty (\Omega ))}+\Vert V_t\Vert _{L^2(L^\infty (\Omega ))} \lesssim \Vert f\Vert _{L^2(L^\infty (\Omega ))}. \end{aligned} \end{aligned}$$

(ii) Let next $\delta \omega _0 = 2 \omega _0$. Then the fundamental matrix is given by

$$\begin{aligned} \begin{aligned} Z(t) =e^{-\omega _0 t} \begin{bmatrix} 1 &  t \\ -\omega _0 \   &  1-\omega _0 t \end{bmatrix}, \quad \left( Z(t) \right) ^{-1} = e^{\omega _0 t} \begin{bmatrix} 1- \omega _0 t &  -t \\ \omega _0 \   &  1 \end{bmatrix}. \end{aligned} \end{aligned}$$

Then

$$\begin{aligned} \begin{aligned} X(t) = e^{-\omega _0 t} \begin{bmatrix} 1 &  t \\ -\omega _0 \   &  1-\omega _0 t \end{bmatrix} \begin{bmatrix} 1 &  0 \\ \omega _0 \   &  1 \end{bmatrix} = e^{-\omega _0 t} \begin{bmatrix} 1+ t\omega _0 \   &  t \\ - \omega _0^2 t \   &  1- \omega _0 t \end{bmatrix} \end{aligned} \end{aligned}$$

and

$$\begin{aligned} \begin{aligned} (X(t))^{-1} = e^{\omega _0 t} \begin{bmatrix} 1- \omega _0 t &  -t \\ \omega _0^2 t \   &  1+ \omega _0 t \end{bmatrix}. \end{aligned} \end{aligned}$$

Then using the method of variation of parameters, we find that the solution has the form

$$\begin{aligned} \begin{aligned} V(t) = X(t)\left( V(0)+ \int _0^t\left( X(\tau )\right) ^{-1}f(\tau )\, d \tau \right) . \end{aligned} \end{aligned}$$

Since periodic solutions must satisfy $V(0)=V(T)$, we then have

$$\begin{aligned} \begin{aligned} (I-X(T)) V(0)= X(T) \int _0^T \left( X(\tau )\right) ^{-1}f(\tau )\, d \tau \end{aligned} \end{aligned}$$

and

$$\begin{aligned} \det (I- X(T)) = (1-e^{-\omega _0 T})^2 > 0, \end{aligned}$$

so we can reason similarly to the first case.

(iii): Lastly, let $\delta \in (0, 2)$. The fundamental matrix is then given by

$$\begin{aligned} \begin{aligned} Z(t) =&\, e^{-\delta \omega _0 t/2} \begin{bmatrix} \cos \alpha t &  \sin \alpha t \\ - \frac{\delta \omega _0}{2} \cos \alpha t - \alpha \sin \alpha t \quad &  -\frac{\delta \omega _0}{2} \sin \alpha t+ \alpha \cos \alpha t \end{bmatrix}. \end{aligned} \end{aligned}$$

We have

$$\begin{aligned} \begin{aligned} \left( Z(0)\right) ^{-1} =&\, \frac{1}{\alpha } \begin{bmatrix} \alpha &  0\\ \frac{\delta \omega _0}{2} \quad & 1 \end{bmatrix}. \end{aligned} \end{aligned}$$

Let $X(t) = Z(t) (Z(0))^{-1}$, we have

$$\begin{aligned} \begin{aligned} X(t) =&\, \frac{1}{\alpha } e^{-\delta \omega _0 t/2} \begin{bmatrix} \cos \alpha t &  \sin \alpha t \\ - \frac{\delta \omega _0}{2} \cos \alpha t - \alpha \sin \alpha t &  -\frac{\delta \omega _0}{2} \sin \alpha t+ \alpha \cos \alpha t \end{bmatrix} \begin{bmatrix} \alpha &  0 \\ \frac{\delta \omega _0}{2} &  1 \end{bmatrix} \\ =&\, \frac{1}{\alpha } e^{- \delta \omega _0 t /2} \begin{bmatrix} \alpha \cos \alpha t + \frac{\delta \omega _0}{2} \sin \alpha t &  \sin \alpha t\\ ( - \alpha ^2 - \frac{\delta ^2 \omega ^2_0}{4} )\sin \alpha t \qquad &  \alpha \cos \alpha t - \frac{\delta \omega _0}{2} \sin \alpha t \end{bmatrix} \end{aligned} \end{aligned}$$

and

$$\begin{aligned} (X(t))^{-1} = \frac{1}{\alpha }e^{\delta \omega _0 t/2} \begin{bmatrix} \alpha \cos \alpha t - \frac{\delta \omega _0}{2} \sin \alpha t &  -\sin \alpha t \\ (\alpha ^2 + \frac{\delta ^2 \omega _0^2}{4})\sin \alpha t \quad &  \alpha \cos \alpha t + \frac{\delta \omega _0}{2} \sin \alpha t \end{bmatrix}. \end{aligned}$$

Then again using the method of variation of parameters, we find that the solution has the form

$$\begin{aligned} \begin{aligned} V(t) = X(t)\left( V(0)+ \int _0^t\left( X(\tau )\right) ^{-1}f(\tau )\, d \tau \right) , \end{aligned} \end{aligned}$$

and

$$\begin{aligned} \det (I- X(T))&= 1+e^{-T \delta \omega _0}-2 e^{-T\frac{\delta \omega _0}{2}}\cos \alpha T = (e^{-T\frac{\delta \omega _0}{2}}-\cos \alpha T)^2+ \sin ^2 \alpha T >0 \end{aligned}$$

since $\delta >0$. Since periodic solutions must satisfy $V(0)=V(T)$, we then have

$$\begin{aligned} \begin{aligned} V(0)= (I-X(T))^{-1}X(T) \int _0^T \left( X(\tau )\right) ^{-1}f(\tau )\, d \tau , \end{aligned} \end{aligned}$$

and arrive at the desired estimate in the same manner as before. $\square$

## B Proof of the Cut-Off Error Estimate (19)

We provide here the proof of the error estimate

19 $$\begin{aligned} \begin{aligned} |\!|\!|(p-\tilde{p}^N, v-\tilde{v}^N)|\!|\!|_{\left( Y^0_p\cap H^2(H^1(\Omega ))\right) \times Y_{v}} \le \, C N^{-\ell } (\Vert p\Vert _{Y_p^{\ell }\cap H^{\ell +2}(0,T; H^1(\Omega ))}+\Vert v\Vert _{Y_v^{\ell }}), \quad \ell \ge 1, \end{aligned} \end{aligned}$$

for $(p, v) \in \left( Y^\ell _p\cap H^{\ell +2}(0,T; H^1(\Omega ))\right) \times Y^\ell _{v}$, which follows by a straightforward modification of the arguments in [3, Lemma 12].

### Proof of (19)

We can express the norms as follows:

and, similarly,

Therefore, the errors are given by

and

We then have the estimate

$$\begin{aligned} \begin{aligned}&\Vert p-\tilde{p}^N\Vert ^2_{Y^0_p\cap H^2(H^1(\Omega ))} \\ \le&\, \frac{T}{2}\tilde{C} \max _{m \ge N+1} \frac{1}{1+(m \omega )^{2\ell }}\sum _{m=N+1}^\infty \Bigl \{ \left( 1+ (m \omega )^2+\ldots +(m \omega )^{2\ell +4}\right) \left( \Vert \nabla p_m^c\Vert ^2_{L^2(\Omega )}+\Vert \nabla p_m^s\Vert ^2_{L^2(\Omega )}\right) \\&\hspace{1cm} +\left( 1+ (m \omega )^2+ (m \omega )^4+\ldots +(m \omega )^{2\ell +4}\right) \left( \Vert p_m^c\Vert ^2_{L^2(\Omega )}+\Vert p_m^s\Vert ^2_{L^2(\Omega )} \right) \\&\hspace{1.5cm} +\left( 1+ (m \omega )^2+ (m \omega )^4+\ldots +(m \omega )^{2\ell +4}\right) \left( \Vert p_m^c\Vert ^2_{L^2(\partial \Omega )}+\Vert p_m^s\Vert ^2_{L^2(\partial \Omega )} \right) \Bigr \} \end{aligned} \end{aligned}$$

for some $\tilde{C}>0$, independent of *N*. From here, we conclude that

$$\begin{aligned} \begin{aligned} \Vert p-\tilde{p}^N\Vert ^2_{Y^0_p\cap H^2(H^1(\Omega ))} \le&\, \frac{T}{2} \tilde{C} \frac{1}{1+((N+1) \omega )^{2\ell }} \Vert p\Vert ^2_{Y_p^\ell \cap H^{\ell +2}(H^1(\Omega ))}\\ \le&\, C N^{-2\ell } \Vert p\Vert ^2_{Y_p^\ell \cap H^{\ell +2}(H^1(\Omega ))}. \end{aligned} \end{aligned}$$

for some $C>0$, independent of *N*. We can analogously derive the estimate

$$\begin{aligned} \begin{aligned} \Vert v-\tilde{v}^N\Vert ^2_{Y^0_p} \le \tilde{C} N^{-2\ell } \Vert v\Vert ^2_{Y_{v}^\ell }, \end{aligned} \end{aligned}$$

to conclude the proof. $\square$

## C Proof of Estimate (22)

We provide here the proof of (22). We start from

38

with the short-hand notation

$$\begin{aligned} f^N= \eta (p^2-(p^{N-1})^2)_{tt} + c^2 \rho _0 n_0 (v_{tt}-v_{tt}^N). \end{aligned}$$

The proof follows by testing (38) with $e^{p,N}$, $e^{p,N}_t$, and $e^{p,N}_{tt}$ and exploiting the time-periodic conditions.

Step I: Testing (38) with $e^{p,N}$ leads to

Above, we have relied on the fact that

$$\begin{aligned} \int _0^T \int _{\Omega } b \nabla e^{p,N}_t \cdot \nabla e^{p,N}\, d xd s=0 \quad \text {and} \quad \int _0^T\int _{\partial \Omega }( c^2 \beta +b \gamma )e^{p,N}_t e^{p,N}\,d \Gamma d s=0 \end{aligned}$$

due to time-periodic conditions. We can further use integration by parts in time together with $e^{p,N}(0)=e^{p,N}(T)$ and $e^{p,N}_t(0)=e^{p,N}_t(T)$ to conclude that

$$\begin{aligned} \begin{aligned}&- \int _0^T \int _{\Omega }e^{p,N}_{tt} e^{p,N}\, d xd s- \int _0^T\int _{\partial \Omega } b \beta e^{p,N}_{tt} e^{p,N}\,d \Gamma d s\\ =&\, \Vert e^{p,N}_{t}\Vert ^2_{L^2(L^2(\Omega ))} + b \beta \Vert e^{p,N}_{t}\Vert ^2_{L^2(L^2(\Omega ))}. \end{aligned} \end{aligned}$$

Furthermore, we have

and thus using Hölder’s and Young’s inequalities leads to

for any $\varepsilon >0$. Similarly,

$$\begin{aligned} \left| -\int _0^T \int _{\Omega }f^Ne^{p,N}\, d xd s\right| \lesssim \Vert f^N\Vert ^2_{L^2(L^2(\Omega ))} +\varepsilon \Vert e^{p,N}\Vert ^2_{L^2(L^2(\Omega ))}. \end{aligned}$$

Thus, for $\varepsilon$ sufficiently small, the outcome of testing in Step I can be formulated as

Further using the equivalence of $\Vert \nabla w\Vert ^2_{L^2(\Omega )}+\Vert w\Vert ^2_{L^2(\partial \Omega )}$ and $\Vert w\Vert ^2_{H^1(\Omega )}$ yields for small $\varepsilon$

Step II: Testing (38) with $e^{p,N}_t$ and exploiting time-periodicity leads to

Then proceeding similarly to Step I results in

$$\begin{aligned} \begin{aligned} \Vert e^{p,N}_t\Vert ^2_{L^2(H^1(\Omega ))} \lesssim \Vert f^N\Vert ^2_{L^2(L^2(\Omega ))}+ \Vert err (\tilde{p}^N)\Vert ^2_{Y^0_p}. \end{aligned} \end{aligned}$$

Step III: Testing (38) with $e^{p,N}_{tt}$ and exploiting time-periodicity leads to

By time-periodicity, we have

$$\begin{aligned} \begin{aligned}&- c^2 \int _0^T \int _{\Omega }\nabla e^{p,N}\cdot \nabla e^{p,N}_{tt} - c^2 \gamma \int _0^T\int _{\partial \Omega } e^{p,N}e^{p,N}_{tt} \,d \Gamma d s\\ =&\, c^2 \Vert \nabla e^{p,N}_t\Vert ^2_{L^2(L^2(\Omega ))} +c^2 \gamma \Vert e^{p,N}_t\Vert ^2_{L^2(L^2(\partial \Omega ))}. \end{aligned} \end{aligned}$$

Furthermore, we have

We integrate by parts in time in the $c^2$ and *b* terms on the left-hand side:

$$\begin{aligned} \begin{aligned}&\int _0^T \int _{\Omega }\left( c^2 \nabla err (\tilde{p}^N)\cdot \nabla e^{p,N}_{tt} + b \nabla (err (\tilde{p}^N))_t \cdot \nabla e^{p,N}_{tt} \right) \, d xd s\\ =&\, -\int _0^T \int _{\Omega }\left( c^2 \nabla (err (\tilde{p}^N))_t \cdot \nabla e^{p,N}_{t} + b \nabla (err (\tilde{p}^N))_{tt} \cdot \nabla e^{p,N}_{t} \right) \, d xd s\end{aligned} \end{aligned}$$

and then estimate

The presence of the term $\Vert \nabla (err (\tilde{p}^N))_{tt}\Vert ^2_{L^2(L^2(\Omega ))}$ above is the reason for assuming $p \in H^{\ell +2}(0,T; H^1(\Omega ))$ (in addition to $p \in \mathcal {X}_p\cap Y^\ell _p$). We also have

$$\begin{aligned} \left| -\int _0^T \int _{\Omega }f^Ne^{p,N}_{tt} \, d xd s\right| \lesssim \Vert f^N\Vert ^2_{L^2(L^2(\Omega ))} + \varepsilon \Vert e^{p,N}_{tt}\Vert ^2_{L^2(L^2(\Omega ))}. \end{aligned}$$

Thus, the outcome of testing in Step III for small enough $\varepsilon$ is

Combining the three obtained estimates leads to (22), provided $\varepsilon$ is sufficiently small.

## Footnotes

1 The program code for the simulations in this section is available as an ancillary file from the arXiv page of this paper.
